# Preparation and in situ use of unstable *N*-alkyl α-diazo-γ-butyrolactams in Rh^II^-catalyzed X–H insertion reactions

**DOI:** 10.3762/bjoc.16.55

**Published:** 2020-04-02

**Authors:** Maria Eremeyeva, Daniil Zhukovsky, Dmitry Dar’in, Mikhail Krasavin

**Affiliations:** 1 Laboratory of Chemical Pharmacology, Institute of Chemistry, Saint Petersburg State University, 26 Universitetskii prospect, Peterhof 198504, Russian Federation

**Keywords:** in situ reactions, *N*-alkyl 2-pyrrolidones, Rh^II^-catalyzed insertion reactions, stability of diazo compounds

## Abstract

*N*-Alkyl α-diazo-γ-butyrolactams, previously found to be unstable and to undergo unproductive dimerization to bishydrazones, were successfully converted immediately to various X–H insertion products with alcohols, aromatic amines and thiols via an in situ Rh^II^-catalyzed reaction. With aliphatic amines or unreactive, sterically hindered anilines, the reactions tended to yield enamine adducts.

## Introduction

**Figure 1:**
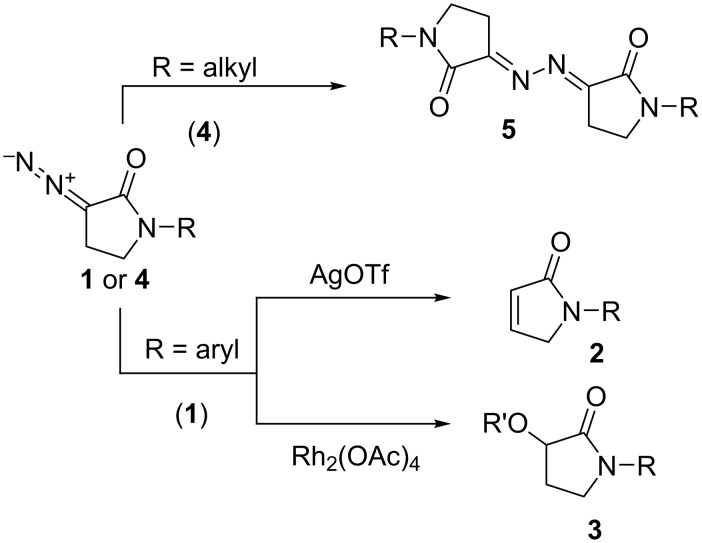
Previously reported uses of α-diazo-γ-butyrolactams **1** and **4**.

Recently, we described the first synthesis and subsequent transformations of a rare type of cyclic α-diazocarbonyl compounds, namely, α-diazo-γ-butyrolactams [1]. In particular, *N*-aryl-α-diazo-γ-butyrolactams **1** were efficiently transformed into pyrrolinones **2** upon the treatment with AgOTf (1 mol %) and into α-alkoxy derivatives **3** via Rh_2_(OAc)_4_-catalyzed O–H insertion reactions with various alcohols. In contrast, *N*-alkyl-α-diazo-γ-butyrolactams **4** did not undergo these reactions typical of α-diazocarbonyl compounds, as they rapidly dimerized to give bishydrazones **5** (Figure 1). The instability of *N*-alkyl-α-diazo-γ-butyrolactams **4** compared to the *N*-aryl counterparts **1**, was most likely related to the reduced electron-withdrawing character of the lactam carbonyl group in the former compared to the latter. This assumption is further supported by the fact that *ortho*-substituted *N*-aryl derivatives **1** (in which the conjugation of the aromatic ring with the lone pair of the lactam nitrogen atom is reduced due to the sterically forced loss of the coplanarity between the aromatic ring and the aminocarbonyl moiety) are as unstable as the *N*-alkyl derivatives **4** [1].

Faced with this serious limitation of the reactivity scope, we set off to investigate the possibility of using unstable compounds **4** in situ, promptly after their formation, in various Rh^II^-catalyzed X–H insertion reactions, particularly the recently described rhodium carbene insertion into O–H [1], N–H [2] and S–H [3] bonds of alcohols, aromatic amines, and thiols, respectively. Herein, we report the results of these studies.

## Results and Discussion

**Scheme 1:**
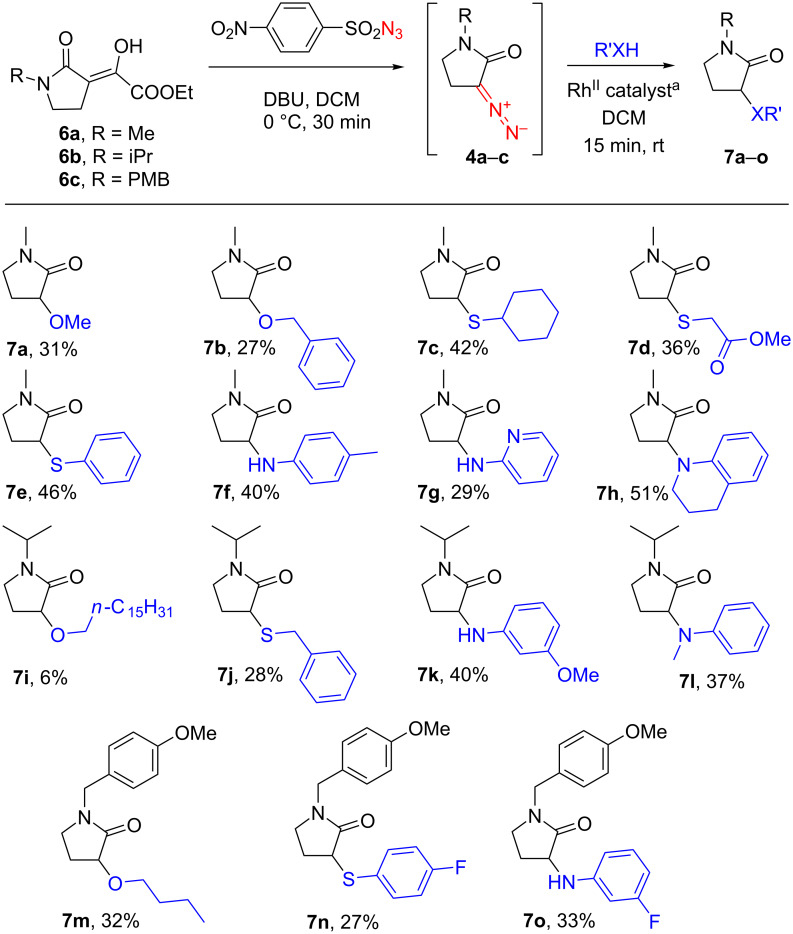
Generation and in situ Rh^II^-catalyzed X–H insertion reactions of the diazo compounds **4a**–**c**. Conditions: ^a^Rh^II^ catalyst: 1 mol % Rh_2_(OAc)_4_ for X = O or S and 0.5 mol % Rh_2_(esp)_2_ for X = NR′′.

Three *N*-alkyl-α-ethoxalyl-γ-lactams **6a**–**c**, prepared by oxalylation of the respective γ-lactams as decribed previously [1], underwent a rapid diazo transfer reaction via the conventional protocol [4–5] employing 4-nitrobenzenesulfonyl azide and DBU. A quick filtration through a plug of alumina (in lieu of silica gel, which led to decomposition of the diazo compounds **4a**–**c**), and addition of an alcohol, a thiol, or an aromatic amine along with a Rh^II^ catalyst resulted in a rapid insertion reaction and the isolation of the desired α-substituted γ-lactams **7a**–**o** in modest yields (Scheme 1). It should be noted that, after some experimentation, the reactions with alcohols and thiols were found to be efficiently catalyzed by 1 mol % of Rh_2_(OAc)_4_ and completed within 30 min. For aromatic amines, this catalyst proved inefficient and was replaced by 0.5 mol % of Rh_2_(esp)_2_. The attempted change of the catalyst to Rh_2_(esp)_2_ in the reactions with alcohols and thiols (which earlier gave a remarkable improvement of the product yields of NH-insertion reactions [2]) resulted in no notable improvement in this case.

**Scheme 2:**
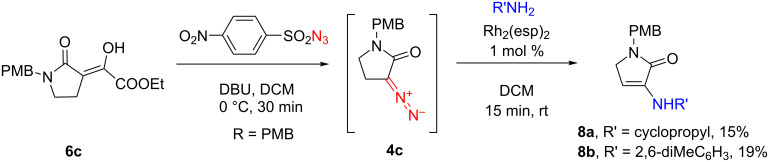
Formation of the enamine coupling products **8a** and **b**.

The only attempt to employ an aliphatic amine, cyclopropylamine (which would presumably be less reactive in the Rh^II^-catalyzed insertion reaction [2]) resulted in the formation of a sole identifiable product – enamine **8a**, isolated chromatographically from a complex mixture of unidentified byproducts. The formation of **8a** (that was also observed previously, along with the expected, saturated coupling product of the Rh^II^-catalyzed reaction of cyclopropylamine with *N*-phenyl-α-diazo-2-pyrrolidone [2]) can be rationalized, as proposed previously [2], either by the oxidation of diazolactam **6c** to a respective ketone (a process described in the literature for other α-diazocarbonyl compounds [6]), followed by a nucleophilic attack of cyclopropylamine. Alternatively, the formation of the enamine product could be envisaged via the reaction of the amine with bishydrazone **5**, which would have formed, if the N–H insertion pathway was not sufficiently rapid. Both assumptions are in line with the formation of the similar enamine coupling product **8b**, that we observed with 2,6-dimethylaniline. With this unreactive, sterically hindered aromatic amine, **6c** is likely to undergo either the unwanted N_2_→O oxidation or dimerize to bishydrazone **5**, whereupon the resulting intermediate would be eventually trapped by the aniline to give **8b** (Scheme 2). The viability of either (or both) of these possibilities is currently investigated. It should be noted that a similar Rh_2_(esp)_2_-catalyzed reaction of one of the *N*-aryl-α-diazo-γ-butyrolactams **1** with 2,6-dimethylaniline previously gave an excellent yield of the N–H insertion product [2].

## Conclusion

We demonstrated that the scope of α-diazo-γ-butyrolactams being capable to undergo Rh^II^-catalyzed X–H insertion reactions with alcohols, thiols, and aromatic amines can be extended to unstable *N*-alkyl derivatives, for which rapid, unproductive dimerization was previously observed. This was achieved through the immediate addition of the X–H insertion partner and a Rh^II^ catalyst to the solution of the diazo compound. The reactions are rapid, albeit moderately yielding. Despite the latter drawback, the range of 1,3-disubstituted 2-pyrrolidones attainable via the intermediate formation of α-diazo-γ-butyrolactams was substantially expanded, thereby making this approach more useful for potential medicinal chemistry exploration of these disubstituted γ-lactams.

## Supporting Information

File 1 General experimental information, synthetic procedures, analytical data and NMR spectra for the reported compounds.
