# Supplementary material for: Preparation and in situ use of unstable N-alkyl α-diazo-γ-butyrolactams in RhII-catalyzed X–H insertion reactions
Source: Beilstein J Org Chem. 2020 Apr 2;16:607–10. doi: 10.3762/bjoc.16.55 (PMC7136545; doi:10.3762/bjoc.16.55)

BEILSTEIN JOURNAL OF ORGANIC CHEMISTRY

Supporting Information

for

Preparation and in situ use of unstable *N*-alkyl α-diazo-γ-butyrolactams in Rh^{II}-catalyzed X–H insertion reactions

Maria Eremeyeva, Daniil Zhukovsky, Dmitry Dar'in and Mikhail Krasavin

Beilstein J. Org. Chem. **2020,** *16,* 607–610. doi:10.3762/bjoc.16.55

General experimental information, synthetic procedures, analytical data and NMR spectra for the reported compounds

License and Terms: This is a supporting information file under the terms of the Creative Commons Attribution License (http://creativecommons.org/licenses/by/4.0). Please note that the reuse, redistribution and reproduction in particular requires that the authors and source are credited.
The license is subject to the *Beilstein Journal of Organic Chemistry* terms and conditions: (https://www.beilstein-journals.org/bjoc)

Table of contents

1. Experimental procedure and characterization data S2

2. Copies of ^{1}H and ^{13}C NMR spectra...S6

1. Experimental procedure and characterization data

General considerations. The acylated γ-butyrolactams **6a–c** were prepared according our previously reported procedure,[S1] other reagents were purchased from commercial sources. Dichloromethane was distilled over CaH_2. Mass spectra were recorded with a Bruker Maxis HRMS-ESI-qTOF spectrometer (electrospray ionization mode). NMR spectroscopic data were recorded with a Bruker Avance 400 spectrometer (400.13 MHz for ^{1}H, 100.61 MHz for ^{13}C and 376.50 MHz for ^{19}F) in $CDCl_3$ and were referenced to residual solvent proton peaks (δ_H = 7.28 ppm) and solvent carbon peaks (δ_C = 77.0 ppm). Melting points were determined with a Stuart SMP50 instrument in open capillary tubes.

General procedure for the X–H insertion reactions (preparation of 7a–o and 8a,b). DBU (61 mg, 0.4 mmol) was added to a solution of acylated lactam **6** (0.4 mmol) and 4-nitrobenzenesulfonyl azide (91 mg, 0.4 mmol) in dichloromethane (5 mL) at 0 °C and the mixture was stirred for 30 min. The resulting solution was filtered through short pad of neutral Al_2O_3, a corresponding alcohol, thiol, or amine (0.5 mmol), and then the appropriate rhodium catalyst (0.004 mmol $Rh_2(OAc)_4$ for O–H and S–H substrates or 0.002 mmol of $Rh_2(esp)_2$ for N–H substrates) was added to the filtrate, and the mixture was stirred for 15 min at room temperature. The solvent was evaporated and the residue was purified using column chromatography employing an ethyl acetate/*n*-hexane 1:3 mixture as the eluent. The same protocol was applied to the reactions that yielded **8a**,**b** (except that the amount of the catalyst was raised to 0.004 mmol).

3-Methoxy-1-methylpyrrolidin-2-one (7a). Yield 16 mg, 31%. Colorless oil. ^{1}H NMR (400 MHz, $CDCl_3$) δ 3.94 (dd, *J* = 7.9, 6.4 Hz, 1H), 3.55 (s, 3H), 3.44 – 3.34 (m, 1H), 3.27 (ddd, *J* = 9.8, 7.8, 6.5 Hz, 1H), 2.87 (d, *J* = 1.7 Hz, 3H), 2.53 – 2.27 (m, 1H), 2.07 – 1.90 (m, 1H). ^{13}C NMR (101 MHz, $CDCl_3$) δ 172.3, 77.9, 57.8, 46.1, 29.8, 25.8. HRMS (ESI/Q-TOF) m/z: $[M+Na]^+$ Calcd for $C_6H_{11}NO_2$ 152.0682; Found 152.0683.

3-(Benzyloxy)-1-methylpyrrolidin-2-one (7b). Yield 22 mg, 27%. Yellow oil. ^{1}H NMR (400 MHz, $CDCl_3$) δ 7.41 (d, *J* = 7.1 Hz, 2H), 7.38 – 7.33 (m, 2H), 7.32 – 7.27 (m, 1H), 4.99 (d, *J* = 11.9 Hz, 1H), 4.77 (d, *J* = 11.9 Hz, 1H), 4.12 (dd, *J* = 8.0, 6.2 Hz, 1H), 3.42 (ddd, *J* = 9.8, 8.6, 4.1 Hz, 1H), 3.25 (ddd, *J* = 9.8, 7.8, 6.4 Hz, 1H), 2.88 (s, 3H), 2.31 (dtd, *J* = 13.3, 7.9, 4.1 Hz, 1H), 2.03 (ddt, *J* = 13.0, 8.7, 6.3 Hz, 1H). ^{13}C NMR (101 MHz, $CDCl_3$) δ 172.7, 138.0, 128.4, 128.0, 127.7, 75.6, 72.0, 46.2, 29.8, 26.3. HRMS (ESI/Q-TOF) m/z: $[M+Na]^+$ Calcd for $C_{12}H_{15}NO_2$ 228.0995; Found 228.1001.

3-(Cyclohexylthio)-1-methylpyrrolidin-2-one (7c). Yield 36 mg, 42%. Orange oil. ^{1}H NMR (400 MHz, $CDCl_3$) δ 3.53 (dd, *J* = 8.8, 4.8 Hz, 1H), 3.46 (ddd, *J* = 9.7, 7.9, 6.5 Hz, 1H), 3.28 (ddd, *J* = 9.7, 8.4, 4.3 Hz, 1H), 3.16 (tt, *J* = 10.3, 3.8 Hz, 1H), 2.86 (s, 3H), 2.45 (dtd, *J* = 13.3, 8.6, 6.6 Hz, 1H), 2.18 – 2.06 (m, 1H), 1.98 – 1.84 (m, 2H), 1.81 – 1.70 (m, 2H), 1.66 – 1.55 (m, 1H), 1.45 – 1.17 (m, 5H). ^{13}C NMR (101 MHz, $CDCl_3$) δ 173.5, 47.4, 43.0, 41.3, 33.8, 33.2, 30.0, 26.8, 26.0, 25.8, 25.8. HRMS (ESI/Q-TOF) m/z: $[M+Na]^+$ Calcd for $C_{11}H_{19}NOS$ 236.1080; Found 236.1085.

Methyl 2-((1-methyl-2-oxopyrrolidin-3-yl)thio)acetate (7d). Yield 29 mg, 36%. Red oil. ^{1}H NMR (400 MHz, $CDCl_3$) δ 3.91 (d, *J* = 15.6 Hz, 1H), 3.74 (s, 3H), 3.67 (dd, *J* = 9.0, 5.3 Hz, 1H), 3.45 (ddd, *J* = 9.7, 8.2, 6.1 Hz, 1H), 3.38 (d, *J* = 15.6 Hz, 1H), 3.31 (ddd, *J* = 9.7, 8.4, 4.7 Hz, 1H), 2.86 (s, 3H), 2.48 (dtd, *J* = 13.8, 8.6, 6.1 Hz, 1H), 1.90 (ddt, *J* = 13.4, 8.1, 5.0 Hz, 1H). ^{13}C NMR (101 MHz, $CDCl_3$) δ 172.8, 170.7, 52.4, 47.3, 42.1, 32.5, 30.0, 25.6. HRMS (ESI/Q-TOF) m/z: $[M+Na]^+$ Calcd for $C_8H_{13}NO_3S$ 226.0508; Found 226.0504.

1-Methyl-3-(phenylthio)pyrrolidin-2-one (7e). Yield 38 mg, 46%. Brown oil. ^{1}H NMR (400 MHz, $CDCl_3$) δ 7.58 – 7.50 (m, 2H), 7.35 – 7.24 (m, 3H), 3.80 (dd, *J* = 9.0, 5.5 Hz, 1H), 3.21 (ddd, *J* = 9.8, 8.4, 4.8 Hz, 1H), 3.06 (ddd, *J* = 9.7, 8.3, 5.8 Hz, 1H), 2.81 (s, 3H), 2.48 (dtd, *J* = 14.3, 8.7, 5.8 Hz, 1H), 2.07 (ddt, *J* = 13.6, 8.2, 5.2 Hz, 1H). ^{13}C NMR (101 MHz, $CDCl_3$) δ 172.1, 133.1, 133.0, 128.9, 128.0, 47.8, 47.3, 30.2, 26.5. HRMS (ESI/Q-TOF) m/z: $[M+Na]^+$ Calcd for $C_{11}H_{13}NOS$ 230.0610; Found 230.0609.

1-Methyl-3-(p-tolylamino)pyrrolidin-2-one (7f). Yield 33 mg, 40%. White solid, mp 111.0-111.9 °C. ^{1}H NMR (400 MHz, $CDCl_3$) δ 7.12 – 6.97 (m, 2H), 6.73 – 6.53 (m, 2H), 4.38 (s, 1H), 3.92 (t, *J* = 8.6 Hz, 1H), 3.57 – 3.29 (m, 2H), 2.95 (s, 3H), 2.71 (dddd, *J* = 12.5, 8.4, 6.7, 1.8 Hz, 1H), 2.27 (s, 3H), 2.08 – 1.81 (m, 1H). ^{13}C NMR (101 MHz, $CDCl_3$) δ 173.2, 145.3, 129.8, 127.5, 113.8, 55.3, 46.4, 30.1, 29.4, 20.4. HRMS (ESI/Q-TOF) m/z: $[M+Na]^+$ Calcd for $C_{12}H_{16}N_2O$ 227.1155; Found 227.1161.

1-Methyl-3-(pyridin-2-ylamino)pyrrolidin-2-one (7g). Yield 22 mg, 29%. White solid, mp 121.5-122.0 °C. ^{1}H NMR (400 MHz, $CDCl_3$) δ 8.09 (ddd, *J* = 5.2, 2.0, 0.9 Hz, 1H), 7.39 (ddd, *J* = 8.6, 7.1, 1.9 Hz, 1H), 6.60 (ddd, *J* = 7.2, 5.1, 1.0 Hz, 1H), 6.54 – 6.44 (m, 1H), 5.14 (d, *J* = 3.9 Hz, 1H), 4.47 – 4.32 (m, 1H), 3.51 – 3.24 (m, 2H), 2.93 (s, 3H), 2.84 (dddd, *J* = 12.5, 8.1, 6.6, 1.5 Hz, 1H), 2.07 – 1.75 (m, 1H). ^{13}C NMR (101 MHz, $CDCl_3$) δ 173.5, 158.1, 147.8, 137.0, 113.5, 109.2, 53.4, 46.5, 30.2, 29.1. HRMS (ESI/Q-TOF) m/z: $[M+Na]^+$ Calcd for $C_{10}H_{13}N_3O$ 214.0951; Found 214.0951.

3-(3,4-Dihydroquinolin-1(2*H*)-yl)-1-methylpyrrolidin-2-one (7h). Yield 47 mg, 51%. White solid, mp 102.9-103.3 °C. ^{1}H NMR (400 MHz, $CDCl_3$) δ 7.10 – 7.01 (m, 1H), 7.00 – 6.96 (m, 1H), 6.69 – 6.58 (m, 2H), 4.60 (t, *J* = 9.1 Hz, 1H), 3.47 – 3.33 (m, 2H), 3.27 (ddd, *J* = 11.0, 6.9, 4.0 Hz, 1H), 3.08 (ddd, *J* = 11.3, 8.2, 3.7 Hz, 1H), 2.94 (s, 3H), 2.79 (t, *J* = 6.5 Hz, 2H), 2.40 (dddd, *J* = 13.1, 8.8, 7.4, 2.6 Hz, 1H), 2.17 – 1.96 (m, 2H), 1.96 – 1.85 (m, 1H). ^{13}C NMR (101 MHz, $CDCl_3$) δ 172.1, 145.1, 129.4, 126.9, 123.9, 116.7, 111.0, 59.4, 46.2, 44.0, 30.4, 28.0, 22.3, 21.2. HRMS (ESI/Q-TOF) m/z: $[M+H]^+$ Calcd for $C_{14}H_{18}N_2O$ 231.1492; Found 231.1491.

3-(Hexadecyloxy)-1-isopropylpyrrolidin-2-one (7i). Yield 9 mg, 6%. White solid, mp 46.3-47.0 °C. ^{1}H NMR (400 MHz, $CDCl_3$) δ 4.38 (hept, *J* = 6.8 Hz, 1H), 4.02 (dd, *J* = 7.8, 6.7 Hz, 1H), 3.89 (dt, *J* = 9.4, 6.8 Hz, 1H), 3.56 (dt, *J* = 9.2, 6.9 Hz, 1H), 3.37 (ddd, *J* = 9.7, 8.5, 3.8 Hz, 1H), 3.19 (dt, *J* = 9.7, 7.2 Hz, 1H), 2.32 (dtd, *J* = 13.1, 7.7, 3.9 Hz, 1H), 1.93 (ddt, *J* = 13.2, 8.6, 6.7 Hz, 1H), 1.70 – 1.57 (m, 2H), 1.38 – 1.24 (m, 26H), 1.17 (d, *J* = 6.8 Hz, 3H), 1.14 (d, *J* = 6.8 Hz, 3H), 0.96 – 0.86 (m, 3H). ^{13}C NMR (101 MHz, $CDCl_3$) δ 171.8, 70.6, 42.5, 38.5, 31.9, 29.9, 29.7 – 29.6 (m), 29.6, 29.6, 29.5, 29.4, 26.5, 26.1, 22.7, 19.7, 19.5, 14.1. HRMS (ESI/Q-TOF) m/z: $[M+Na]^+$ Calcd for $C_{23}H_{45}NO_2$ 390.3343; Found 390.3345.

3-(Benzylthio)-1-isopropylpyrrolidin-2-one (7j). Yield 28 mg, 28%. Orange oil. ^{1}H NMR (400 MHz, $CDCl_3$) δ 7.49 – 7.39 (m, 2H), 7.36 – 7.30 (m, 2H), 7.28 – 7.22 (m, 1H), 4.38 (hept, *J* = 6.8 Hz, 1H), 4.21 (d, *J* = 13.2 Hz, 1H), 3.87 (d, *J* = 13.2 Hz, 1H), 3.44 – 3.29 (m, 2H), 3.24 (ddd, *J* = 9.5, 8.2, 4.2 Hz, 1H), 2.31 (dtd, *J* = 13.4, 8.5, 6.7 Hz, 1H), 1.95 – 1.74 (m, 1H), 1.29 – 1.08 (m, 6H). ^{13}C NMR (101 MHz, $CDCl_3$) δ 172.6, 138.1, 129.2, 128.5, 127.0, 42.8, 42.2, 39.9, 35.3, 26.1, 19.8, 19.5. HRMS (ESI/Q-TOF) m/z: $[M+H]^+$ Calcd for $C_{14}H_{19}NOS$ 250.1260; Found 250.1266.

1-Isopropyl-3-((3-methoxyphenyl)amino)pyrrolidin-2-one (7k). Yield 40 mg, 40%. Brown solid, mp 123.7-124.7 °C. ^{1}H NMR (400 MHz, $CDCl_3$) δ 7.12 (t, *J* = 8.1 Hz, 1H), 6.35 (dd, *J* = 8.1, 2.3 Hz, 1H), 6.30 (dd, *J* = 8.0, 2.2 Hz, 1H), 6.23 (t, *J* = 2.3 Hz, 1H), 4.43 (hept, *J* = 6.8 Hz, 1H), 3.94 (dd, *J* = 9.8, 7.8 Hz, 1H), 3.79 (s, 3H), 3.50 – 3.22 (m, 2H), 2.89 – 2.59 (m, 1H), 1.92 – 1.74 (m, 1H), 1.25 – 0.98 (m, 6H). ^{13}C NMR (101 MHz, $CDCl_3$) δ 172.1, 160.8, 149.0, 130.0,

106.7, 103.3, 99.8, 55.8, 55.1, 43.2, 38.9, 29.5, 19.8, 19.6. HRMS (ESI/Q-TOF) m/z: $[M+H]^+$ Calcd for $C_{14}H_{20}N_2O_2$ 249.1598; Found 249.1601.

1-Isopropyl-3-(methyl(phenyl)amino)pyrrolidin-2-one (7l). Yield 34 mg, 37%. Brown amorphous solid. ^{1}H NMR (400 MHz, $CDCl_3$) δ 7.28 – 7.21 (m, 2H), 6.84 (d, *J* = 8.2 Hz, 2H), 6.77 (t, *J* = 7.3 Hz, 1H), 4.61 (t, *J* = 9.1 Hz, 1H), 4.49 (hept, *J* = 6.6 Hz, 1H), 3.39 (td, *J* = 9.5, 2.3 Hz, 1H), 3.33 – 3.23 (m, 1H), 2.85 (s, 3H), 2.49 – 2.28 (m, 1H), 2.08 – 1.85 (m, 1H), 1.22 (d, *J* = 6.8 Hz, 3H), 1.18 (d, *J* = 6.8 Hz, 3H). ^{13}C NMR (101 MHz, $CDCl_3$) δ 171.3, 149.8, 129.1, 117.7, 113.8, 61.8, 43.0, 38.4, 33.8, 21.6, 19.7, 19.6. HRMS (ESI/Q-TOF) m/z: $[M+H]^+$ Calcd for $C_{14}H_{20}N_2O$ 233.1648; Found 233.1653.

3-Butoxy-1-(4-methoxybenzyl)pyrrolidin-2-one (7m). Yield 36 mg, 32%. Orange oil. ^{1}H NMR (400 MHz, $CDCl_3$) δ 7.19 – 7.15 (m, 2H), 6.89 – 6.82 (m, 2H), 4.40 (d, *J* = 2.0 Hz, 2H), 4.07 (dd, *J* = 7.8, 6.6 Hz, 1H), 3.92 (dt, *J* = 9.2, 6.7 Hz, 1H), 3.81 (s, 3H), 3.59 (dt, *J* = 9.2, 6.7 Hz, 1H), 3.27 (ddd, *J* = 9.8, 8.6, 3.9 Hz, 1H), 3.12 (ddd, *J* = 9.8, 7.6, 6.6 Hz, 1H), 2.30 (dtd, *J* = 13.1, 7.7, 3.9 Hz, 1H), 1.92 (ddt, *J* = 13.2, 8.6, 6.6 Hz, 1H), 1.71 – 1.55 (m, 2H), 1.49 – 1.35 (m, 2H), 0.94 (t, *J* = 7.4 Hz, 3H). ^{13}C NMR (101 MHz, $CDCl_3$) δ 172.5, 159.2, 129.5, 128.2, 114.1, 76.9, 70.3, 55.3, 46.1, 43.2, 31.9, 26.3, 19.3, 13.9. HRMS (ESI/Q-TOF) m/z: $[M+Na]^+$ Calcd for $C_{16}H_{23}NO_3$ 300.1570; Found 300.1560.

3-((4-Fluorophenyl)thio)-1-(4-methoxybenzyl)pyrrolidin-2-one (7n). Yield 35 mg, 27%. Yellow solid, mp 99.0-99.8 °C. ^{1}H NMR (400 MHz, $CDCl_3$) δ 7.73 – 7.53 (m, 2H), 7.19 – 7.06 (m, 2H), 7.05 – 6.96 (m, 2H), 6.90 – 6.79 (m, 2H), 4.42 (d, *J* = 14.5 Hz, 1H), 4.30 (d, *J* = 14.5 Hz, 1H), 3.81 (s, 3H), 3.78 (dd, *J* = 9.1, 6.0 Hz, 1H), 3.12 (ddd, *J* = 9.9, 8.3, 5.3 Hz, 1H), 3.01 (ddd, *J* = 9.8, 8.4, 5.5 Hz, 1H), 2.44 (dtd, *J* = 13.9, 8.6, 5.4 Hz, 1H), 2.01 (ddt, *J* = 13.8, 8.4, 5.6 Hz, 1H). ^{13}C NMR (101 MHz, $CDCl_3$) δ 171.7, 163.0 (d, *J* = 248.7 Hz), 159.2, 136.3 (d, *J* = 8.4 Hz), 129.5, 128.0, 127.7 (d, *J* = 3.4 Hz), 116.0 (d, *J* = 21.7 Hz), 114.1, 55.3, 48.4, 46.6, 44.3, 26.0. ^{19}F NMR (376 MHz, $CDCl_3$) δ -112.9. HRMS (ESI/Q-TOF) m/z: $[M+H]^+$ Calcd for $C_{18}H_{18}FNO_2S$ 332.1115; Found 332.1115.

3-((3-Fluorophenyl)amino)-1-(4-methoxybenzyl)pyrrolidin-2-one (7o). Yield 41 mg, 33%. Brown solid, mp 99.3-101.2 °C. ^{1}H NMR (400 MHz, $CDCl_3$) δ 7.24 – 7.18 (m, 2H), 7.13 (td, *J* = 8.2, 6.6 Hz, 1H), 6.95 – 6.86 (m, 2H), 6.51 – 6.41 (m, 2H), 6.36 (dt, *J* = 11.3, 2.3 Hz, 1H), 4.68 (s, 1H), 4.51 (d, *J* = 14.5 Hz, 1H), 4.45 (d, *J* = 14.5 Hz, 1H), 4.00 (dd, *J* = 9.7, 7.9 Hz, 1H), 3.82 (s, 3H), 3.41 – 3.16 (m, 2H), 2.67 (dddd, *J* = 13.4, 8.2, 5.5, 2.8 Hz, 1H), 1.97 – 1.74 (m, 1H). ^{13}C NMR (101 MHz, $CDCl_3$) δ 172.5, 164.0 (d, *J* = 243.1 Hz), 159.3, 149.3 (d, *J* = 10.5 Hz), 130.4 (d, *J* = 10.1 Hz), 129.5, 127.9, 114.2, 109.6 (d, *J* = 2.4 Hz), 104.7 (d, *J* = 21.5 Hz), 100.3 (d, *J* = 25.3 Hz), 55.3, 55.3, 46.6, 43.4, 29.1. ^{19}F NMR (376 MHz, $CDCl_3$) δ -112.7. HRMS (ESI/Q-TOF) m/z: $[M+Na]^+$ Calcd for $C_{18}H_{19}FN_2O_2$ 337.1323; Found 337.1322.

3-(Cyclopropylamino)-1-(4-methoxybenzyl)-1,5-dihydro-2*H*-pyrrol-2-one (8a). Yield 15 mg, 15%. Orange oil. ^{1}H NMR (400 MHz, $CDCl_3$) δ 7.26 – 7.21 (m, 2H), 7.12 – 7.01 (m, 3H), 6.94 – 6.86 (m, 2H), 5.66 (s, 1H), 4.88 (t, *J* = 2.4 Hz, 1H), 4.65 (s, 2H), 3.82 (s, 3H), 3.70 (d, *J* = 2.4 Hz, 2H), 2.26 (s, 6H). ^{13}C NMR (101 MHz, $CDCl_3$) δ 167.7, 159.0, 140.4, 129.4, 129.3, 114.0, 98.7, 55.3, 48.5, 46.0, 25.3, 6.4. HRMS (ESI/Q-TOF) m/z: $[M+Na]^+$ Calcd for $C_{15}H_{18}N_2O_2$ 281.1260; Found 281.1270.

3-((2,6-Dimethylphenyl)amino)-1-(4-methoxybenzyl)-1,5-dihydro-2*H*-pyrrol-2-one (8b).

Yield 24 mg, 19%. Yellow oil. ^{1}H NMR (400 MHz, $CDCl_3$) δ 7.26 – 7.21 (m, 2H), 7.12 – 7.01 (m, 3H), 6.94 – 6.86 (m, 2H), 5.66 (s, 1H), 4.88 (t, *J* = 2.4 Hz, 1H), 4.63a5 (s, 2H), 3.82 (s, 3H),

3.70 (d, J = 2.4 Hz, 2H), 2.26 (s, 6H). ^{13}C NMR (101 MHz, $CDCl_3$) δ 167.5, 159.1, 138.1, 137.3, 134.6, 129.5, 129.3, 128.2, 125.8, 114.1, 99.4, 55.3, 48.3, 46.2, 18.2. HRMS (ESI/Q-TOF) m/z: $[M+Na]^+$ Calcd for $C_{20}H_{22}N_2O_2$ 345.1573; Found 345.1577.

Reference

[S1] D. Zhukovsky, D. Dar'in, G. Kantin, M. Krasavin, *Eur. J. Org. Chem.* **2019**, *2019*, 2397.

2. Copies of ^{1}H and ^{13}C NMR spectra

^{1}H and ^{13}C NMR spectra of compound 7a

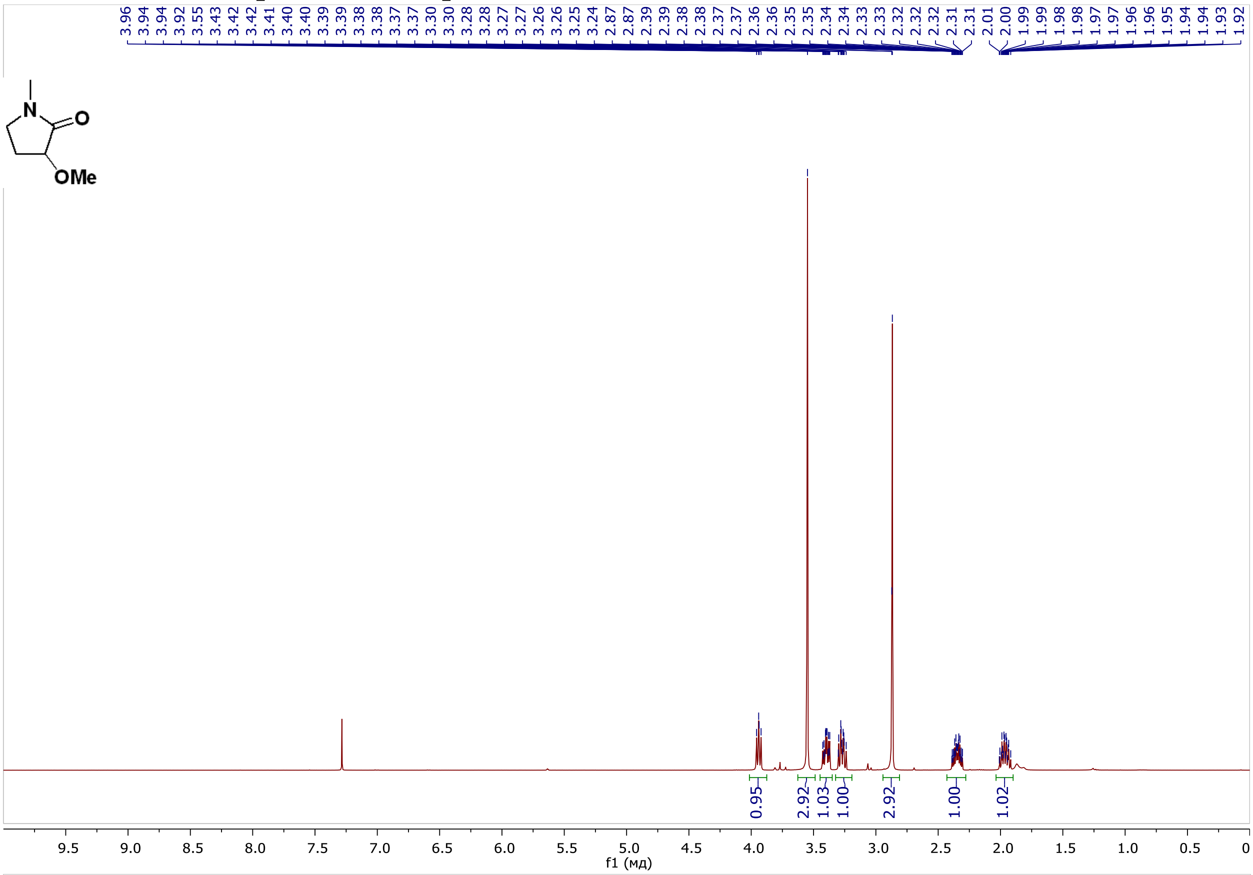

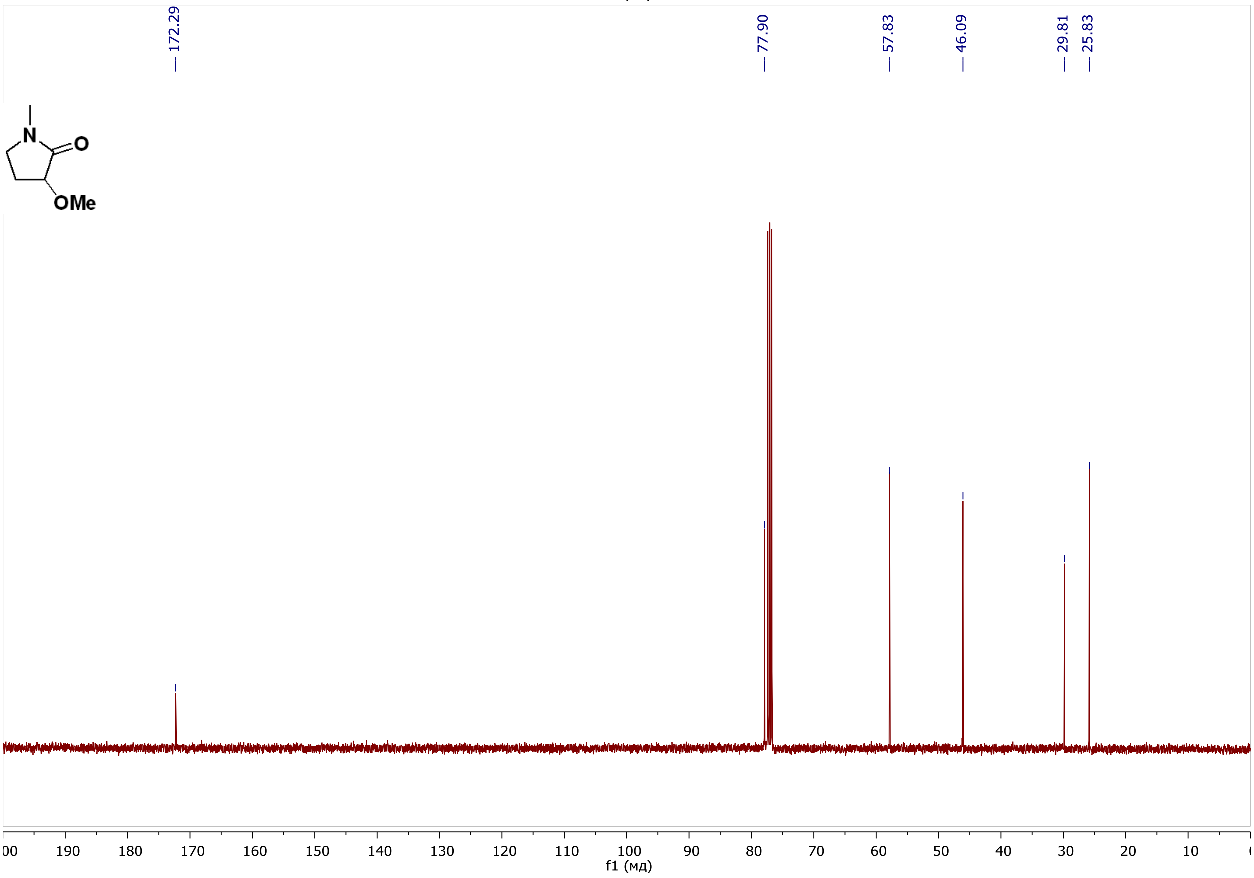

^{1}H and ^{13}C NMR spectra of compound 7b

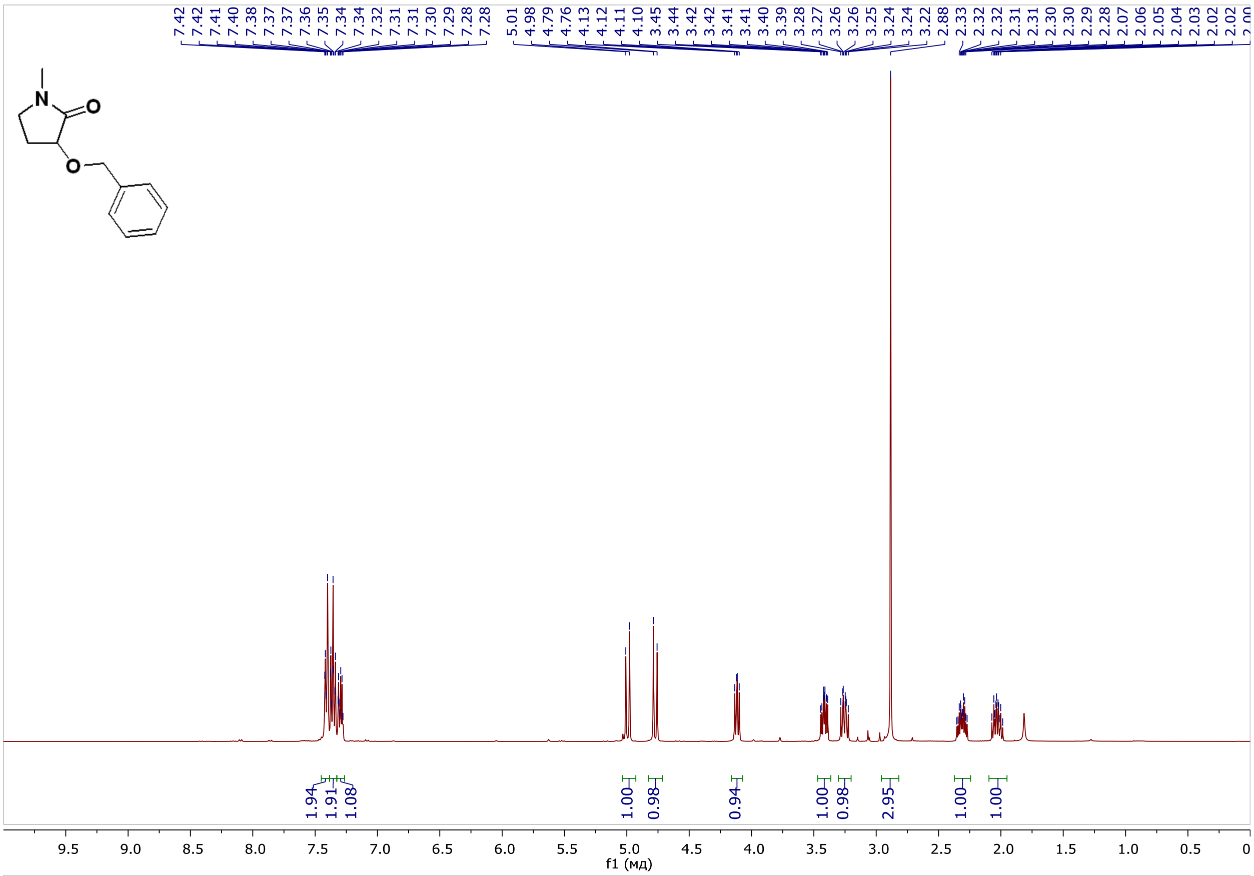

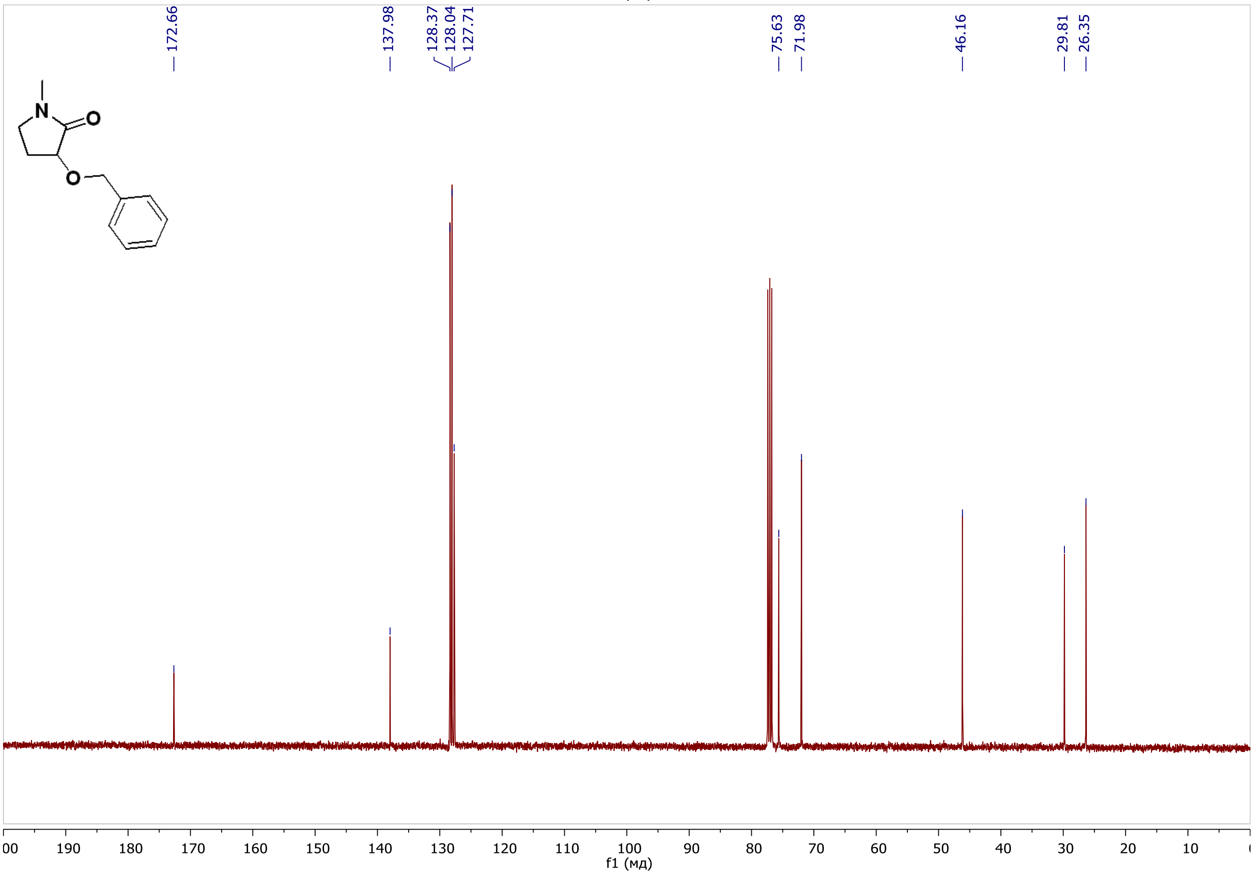

^{1}H and ^{13}C NMR spectra of compound 7c

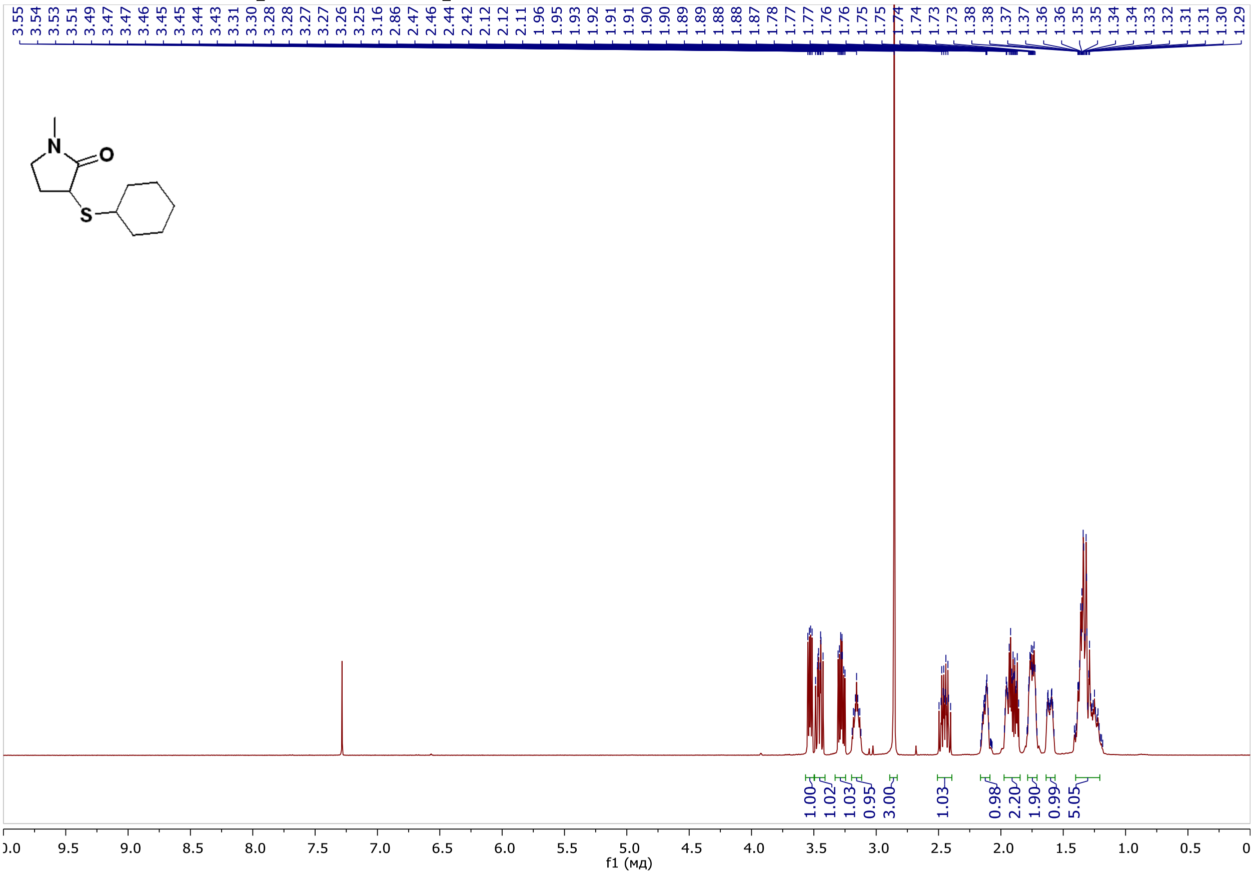

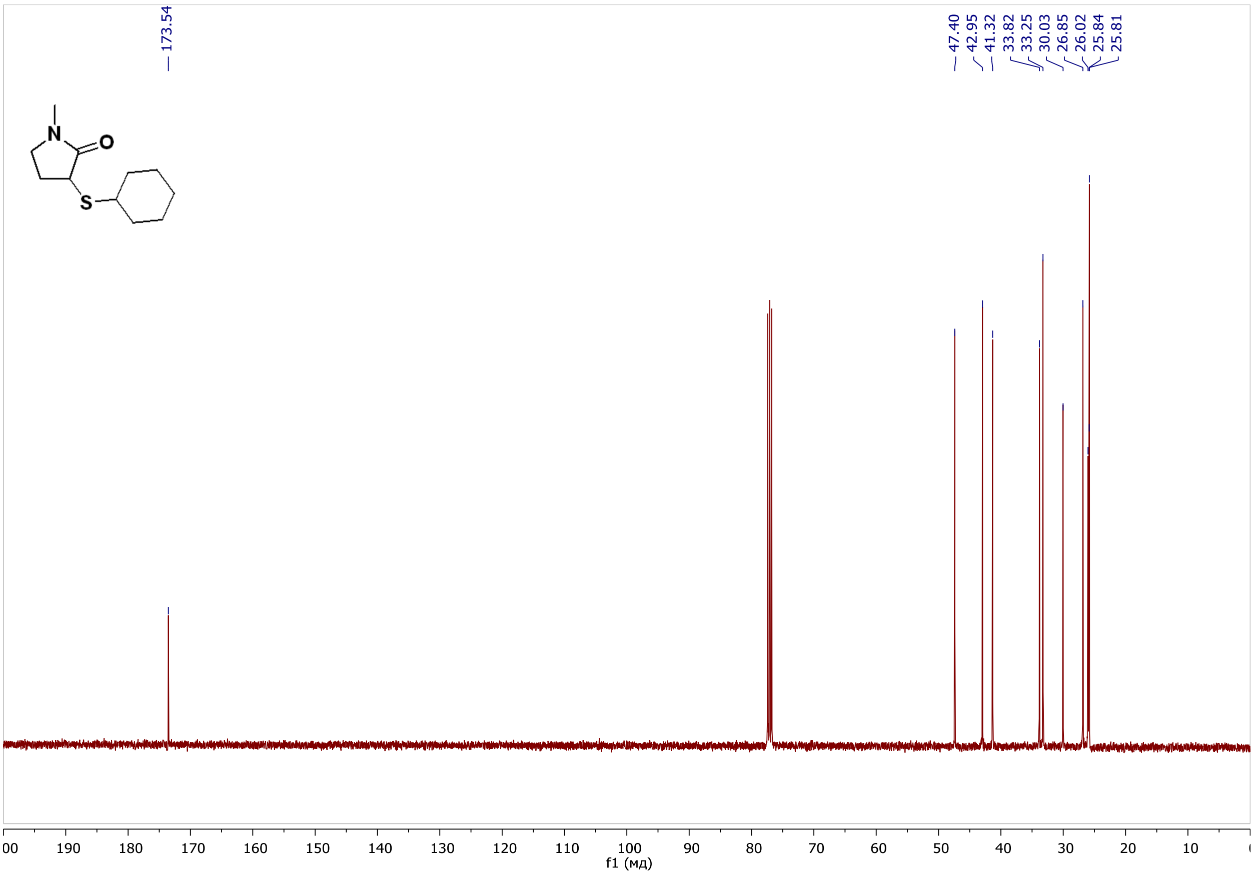

^{1}H and ^{13}C NMR spectra of compound 7d

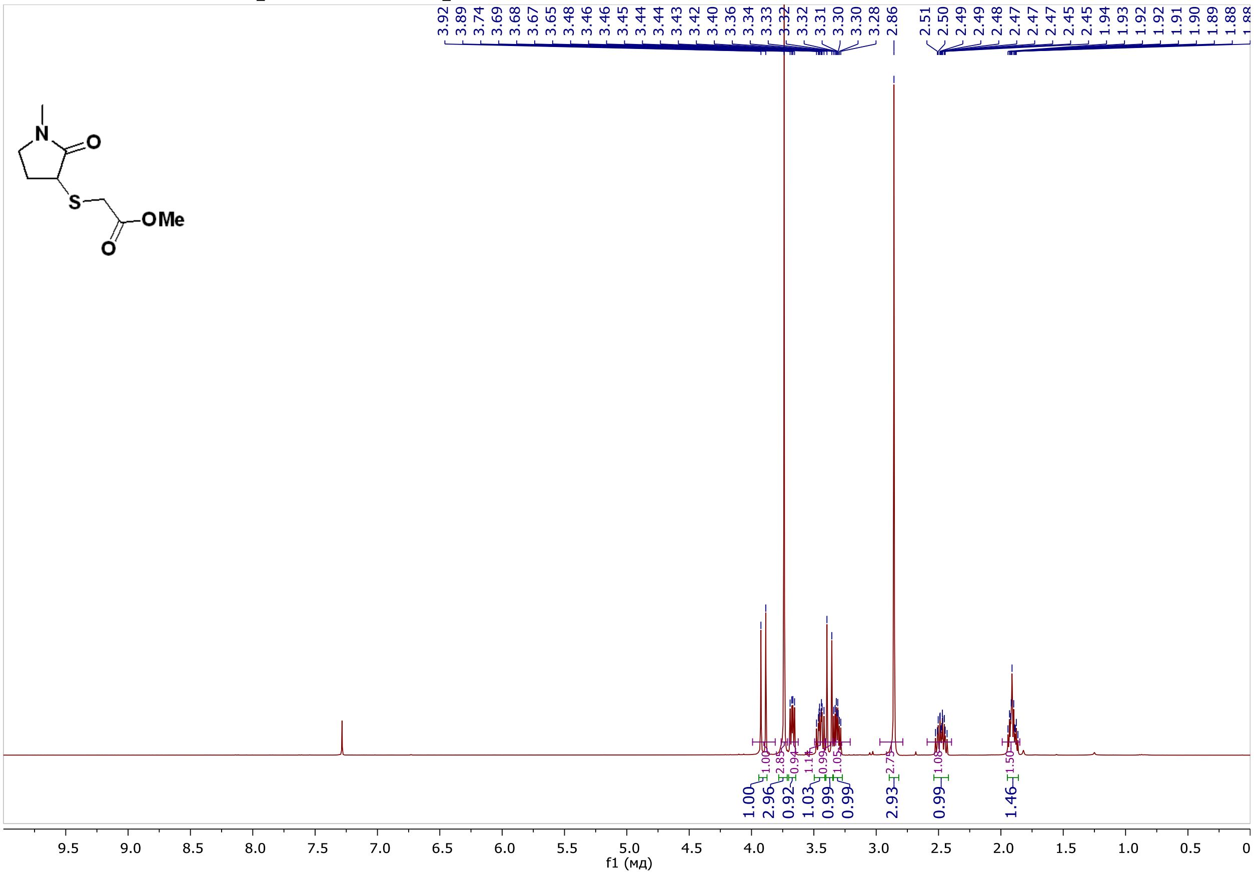

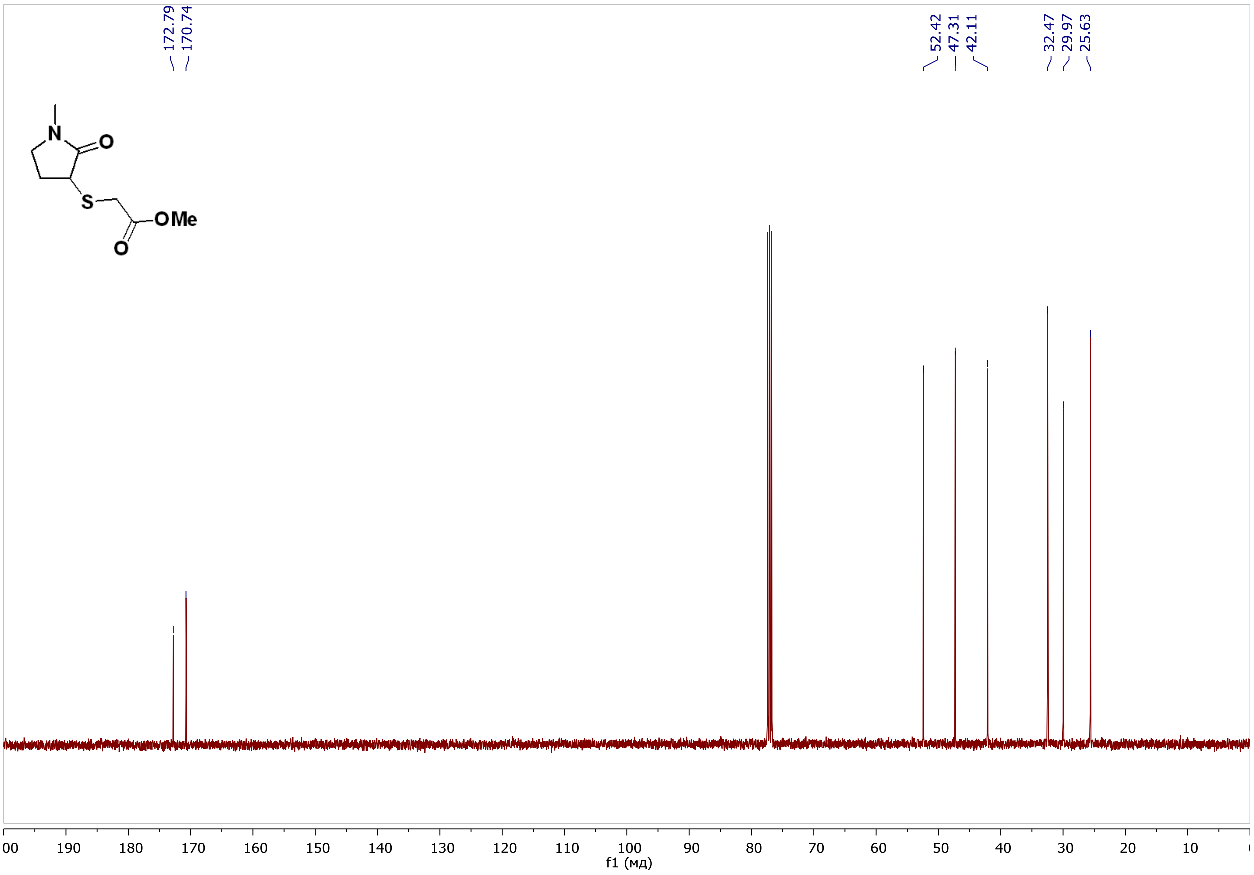

^{1}H and ^{13}C NMR spectra of compound 7e

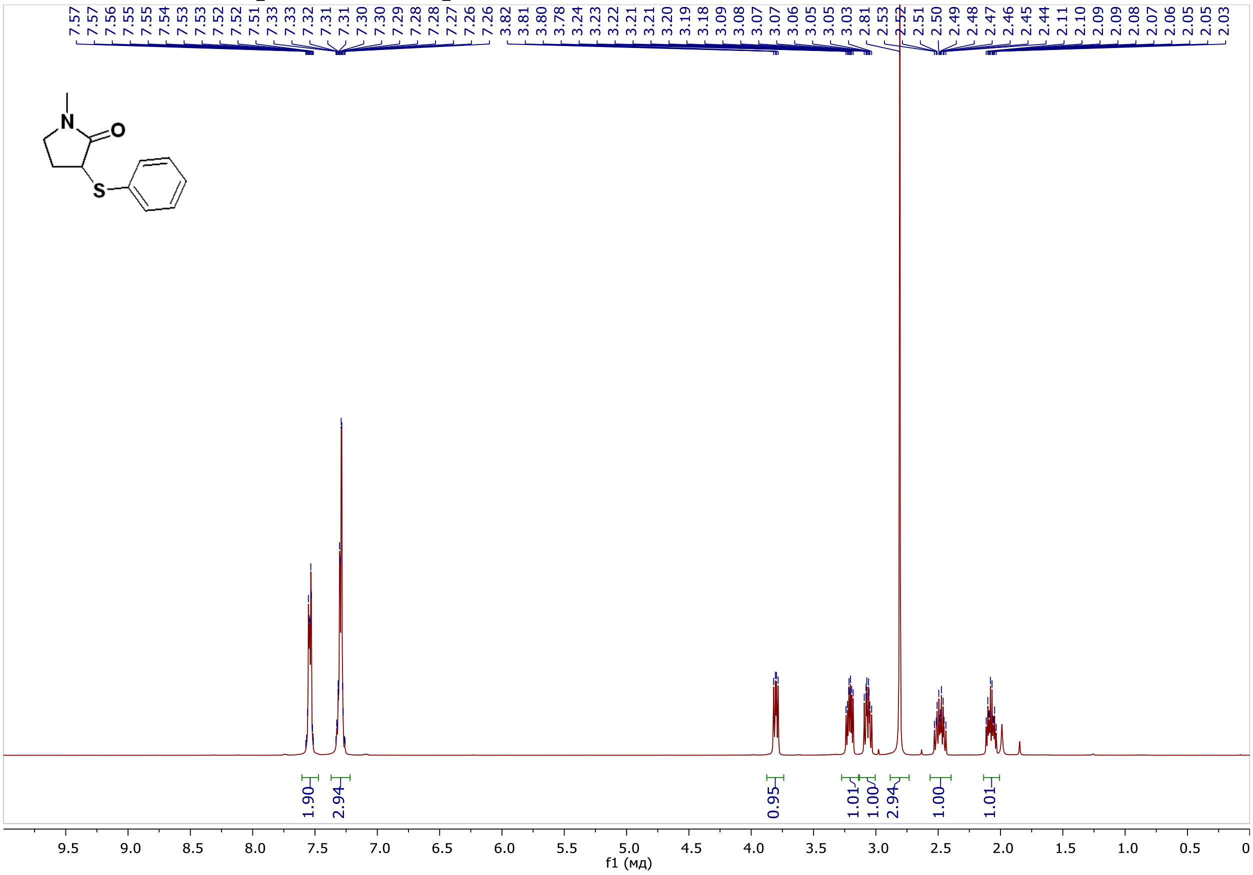

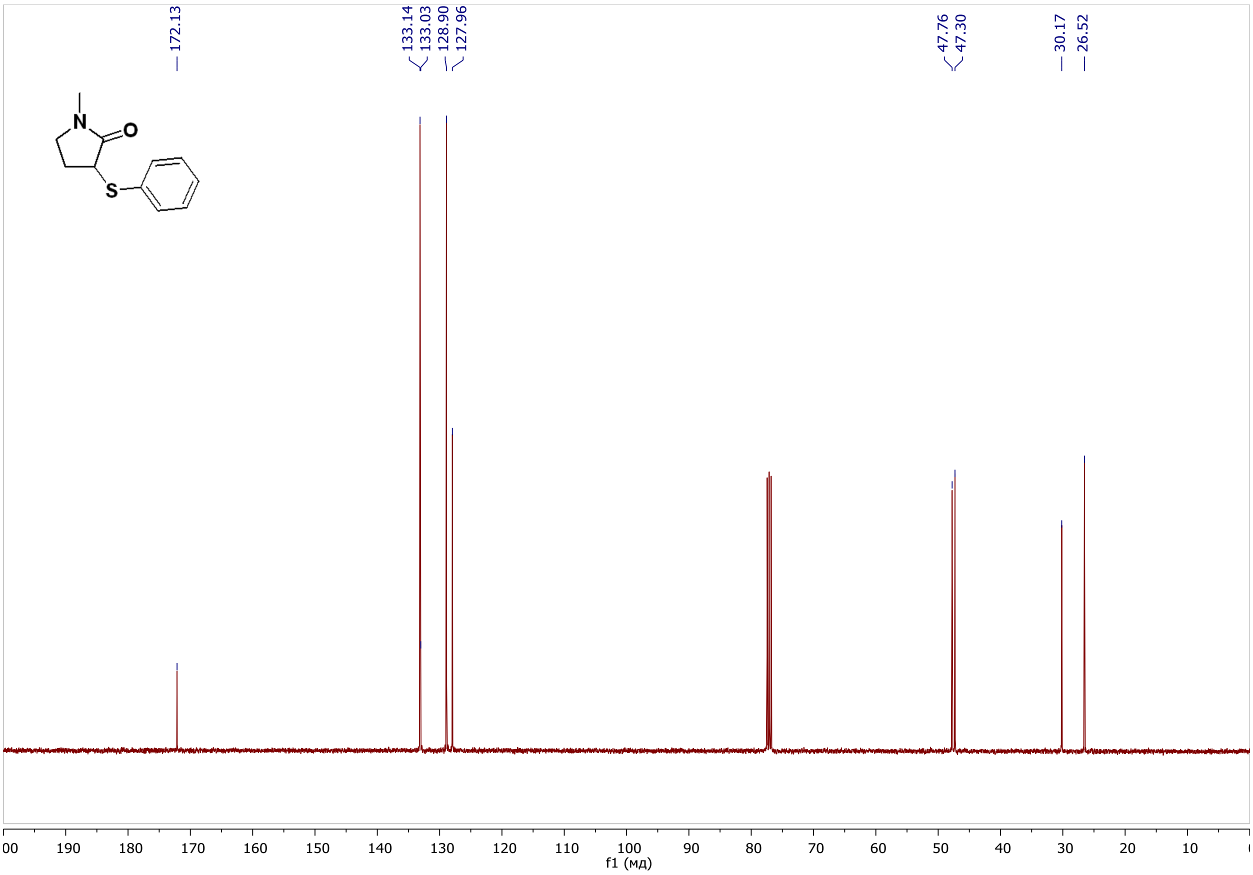

^{1}H and ^{13}C NMR spectra of compound 7f

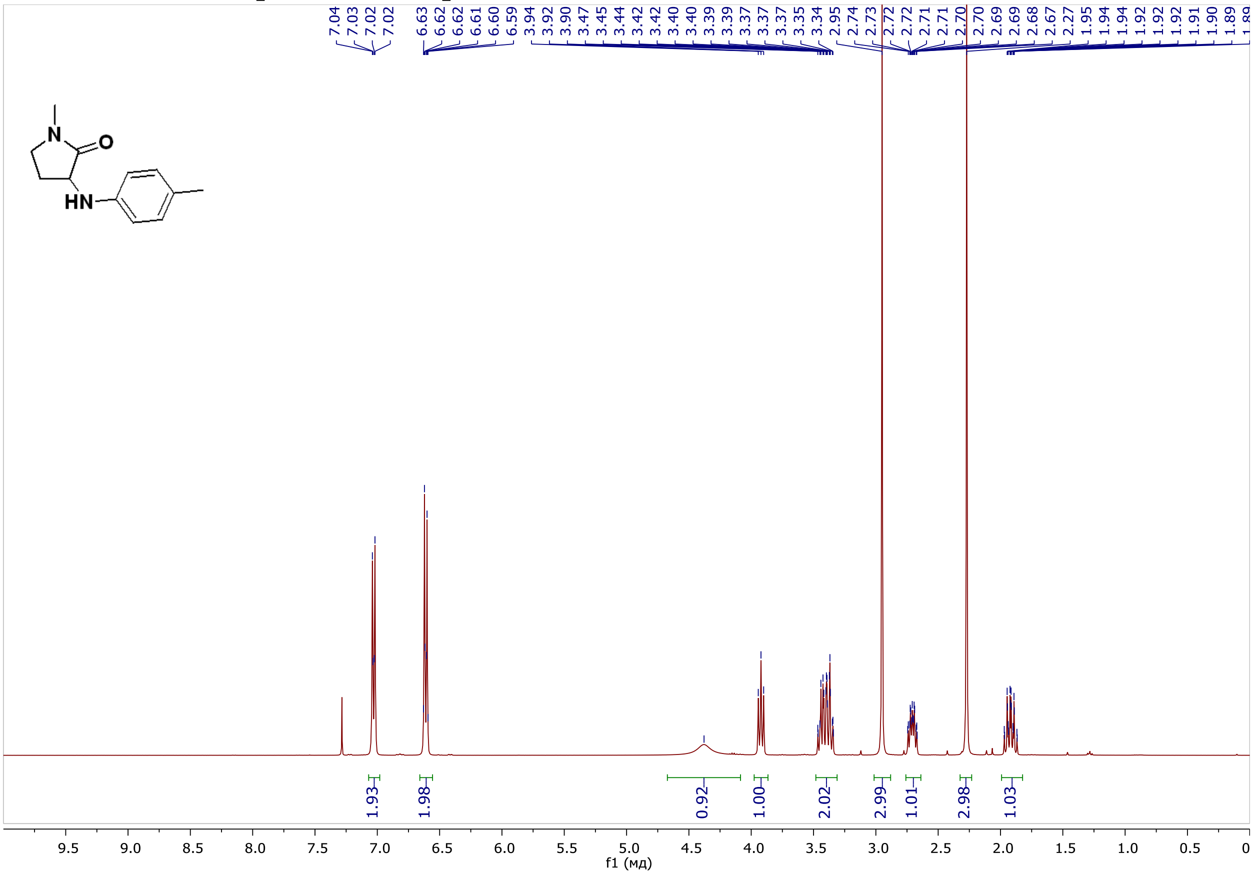

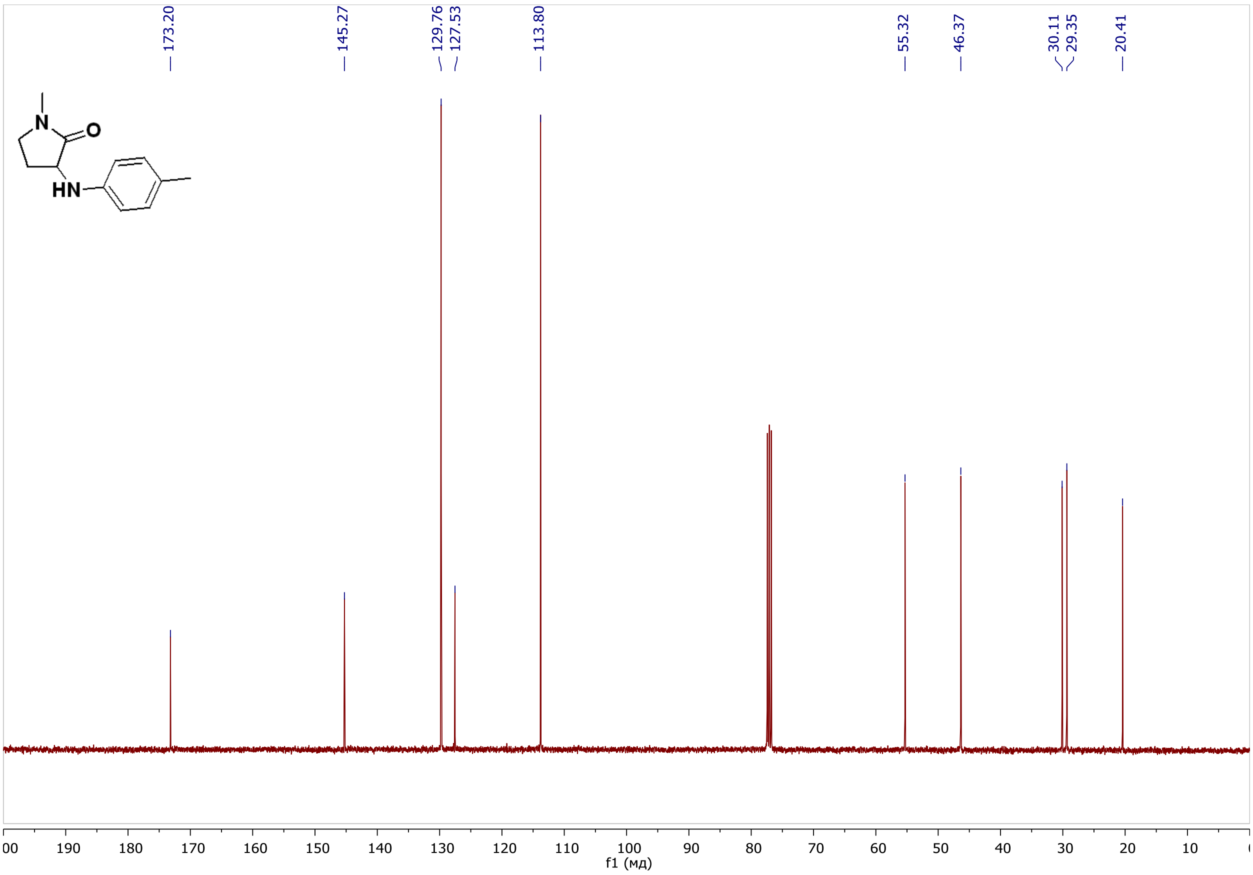

^{1}H and ^{13}C NMR spectra of compound 7g

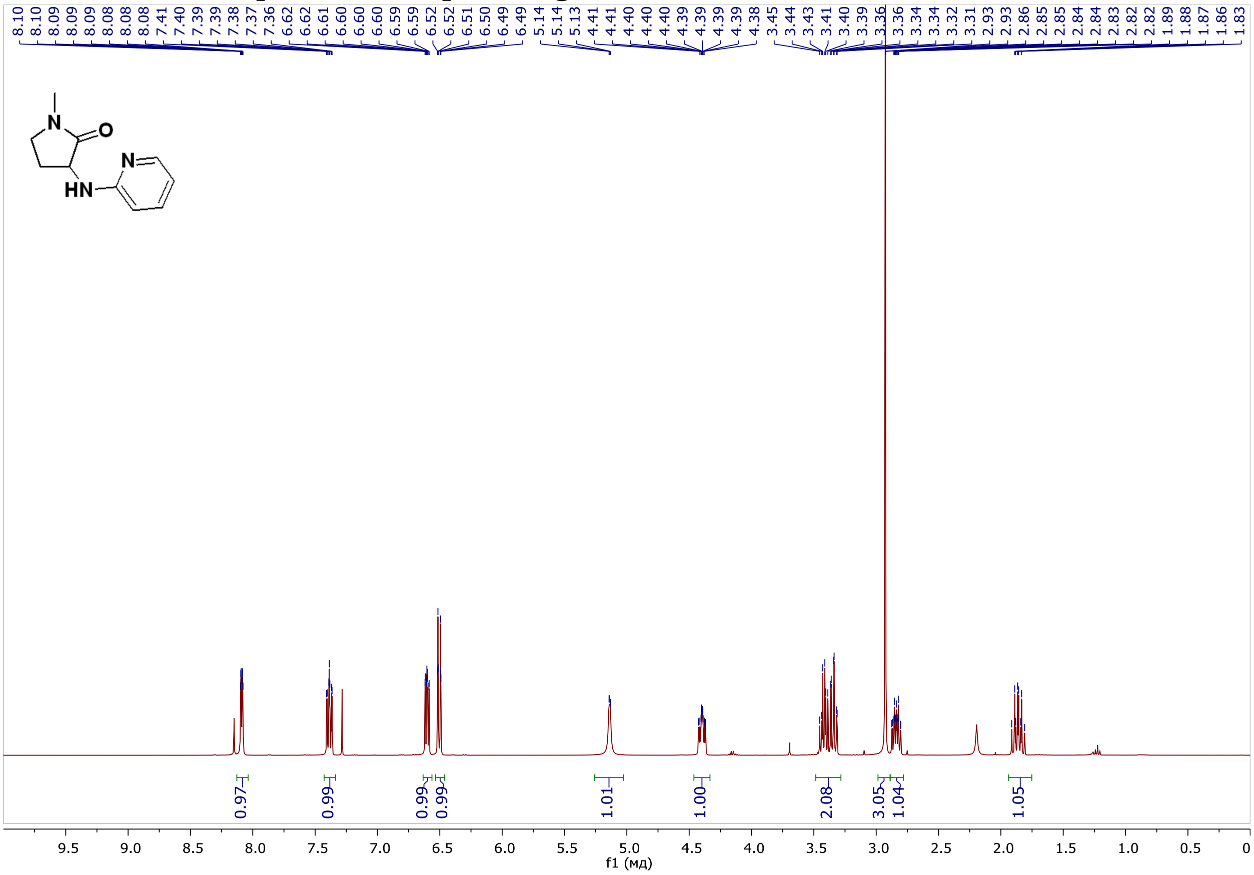

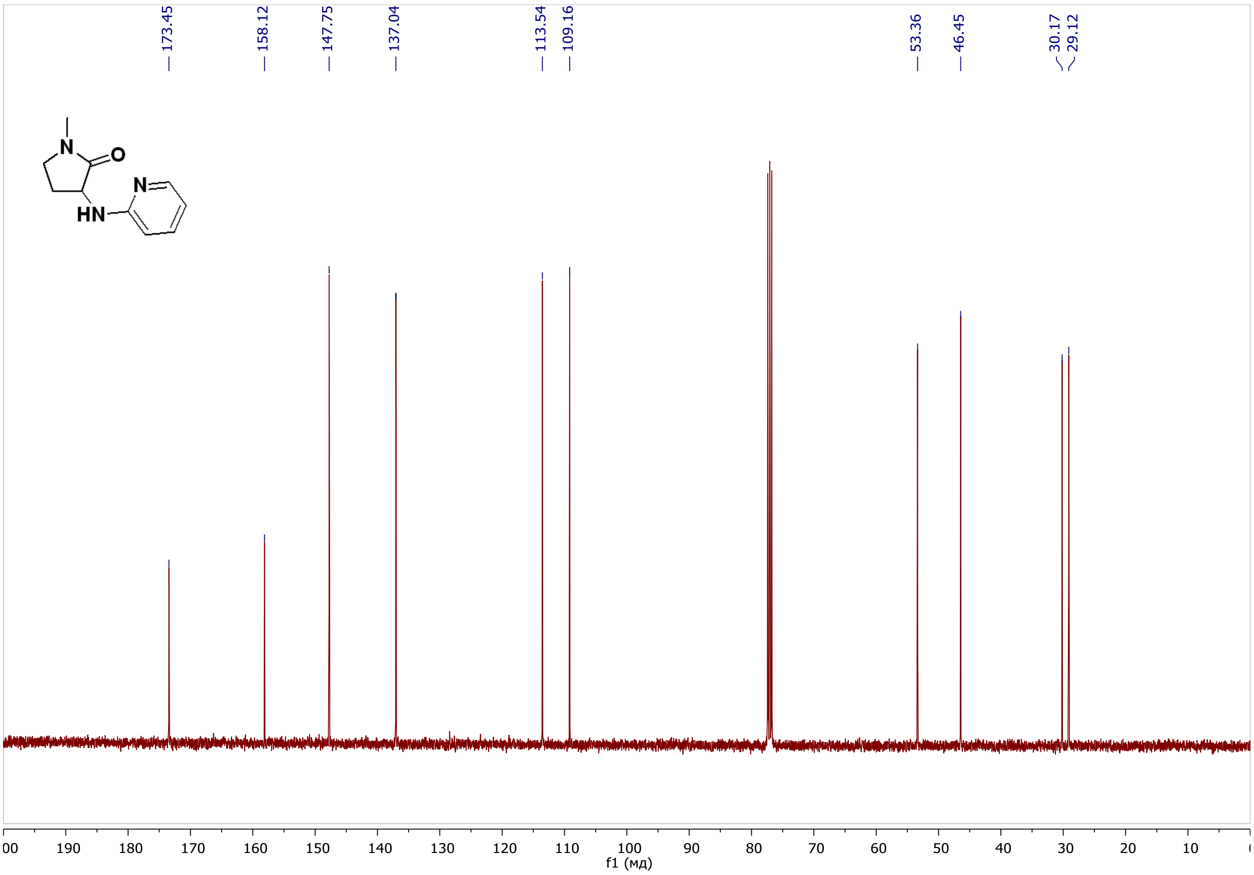

^{1}H and ^{13}C NMR spectra of compound 7h

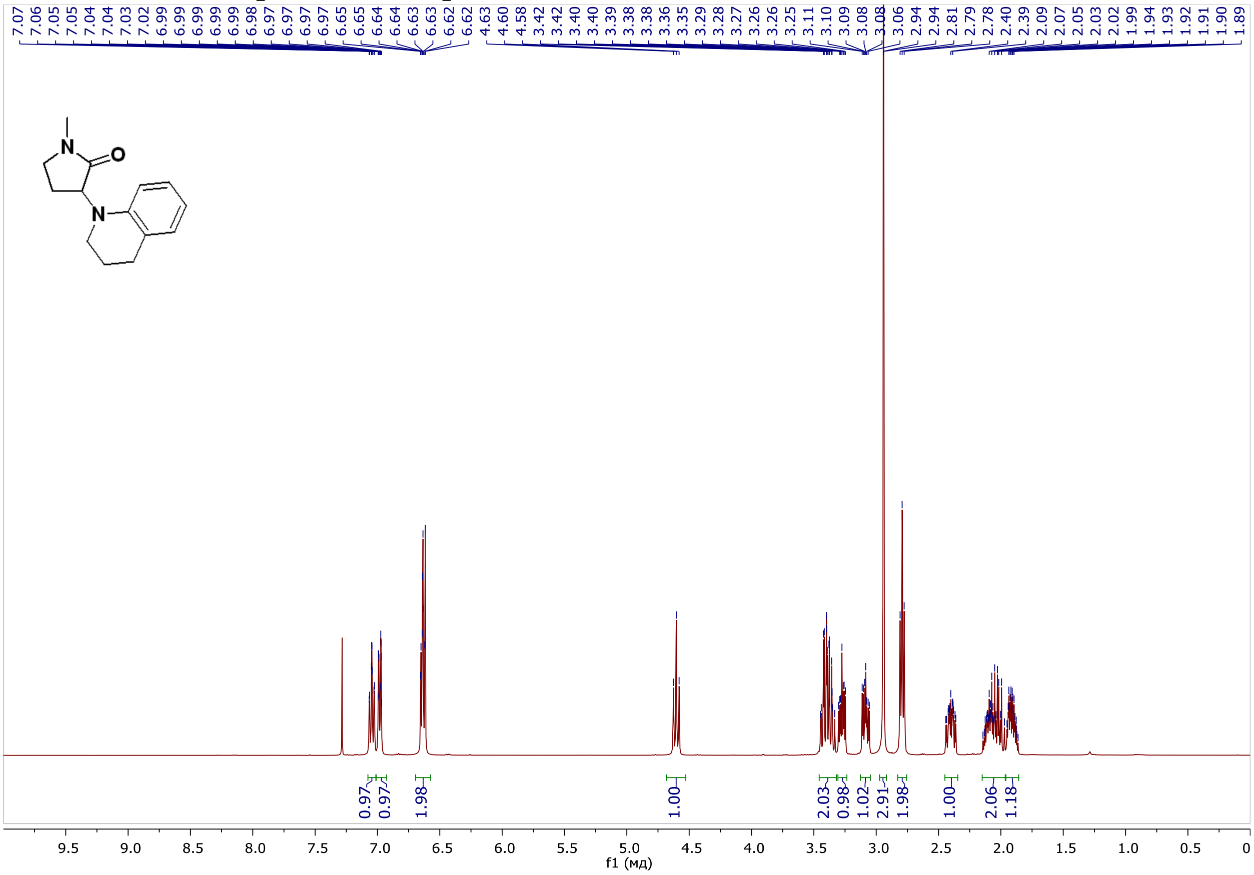

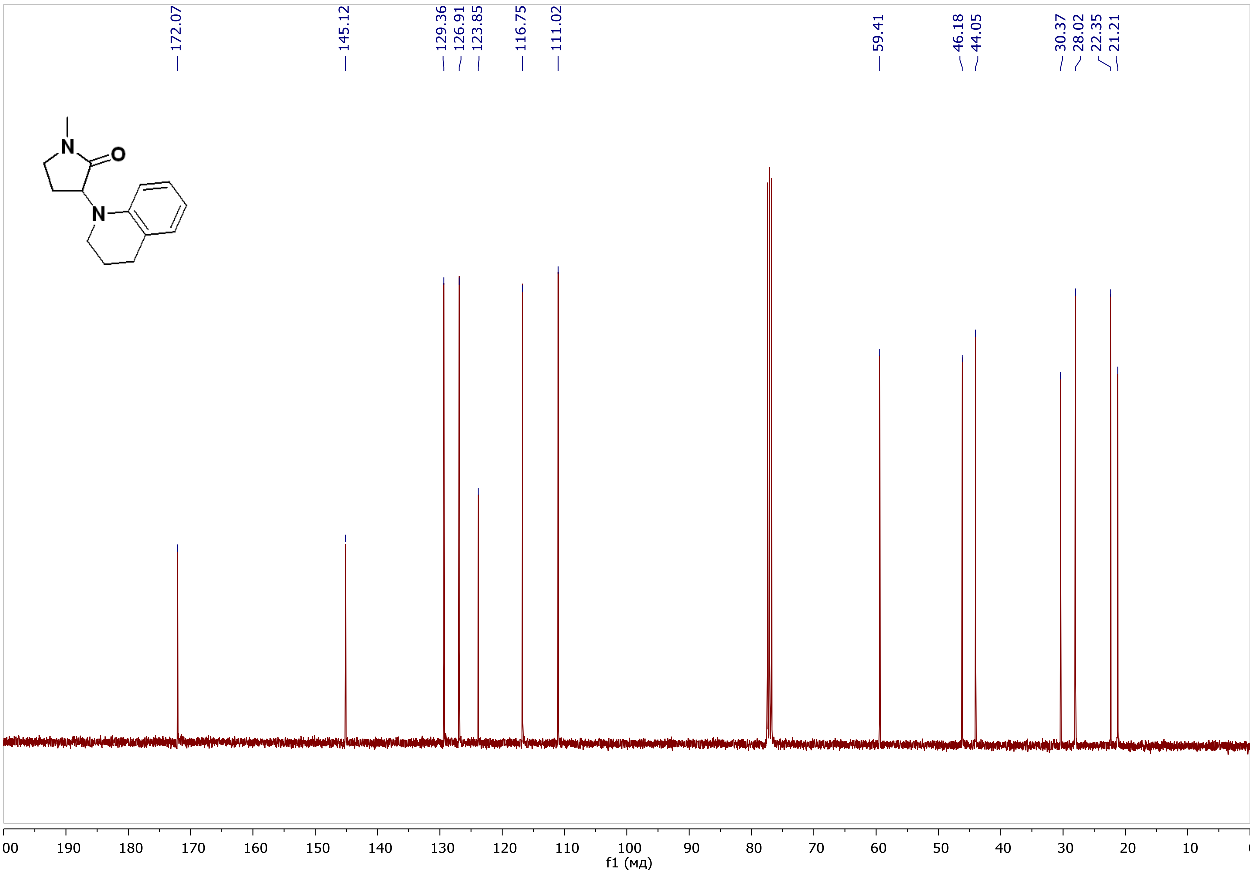

^{1}H and ^{13}C NMR spectra of compound 7i

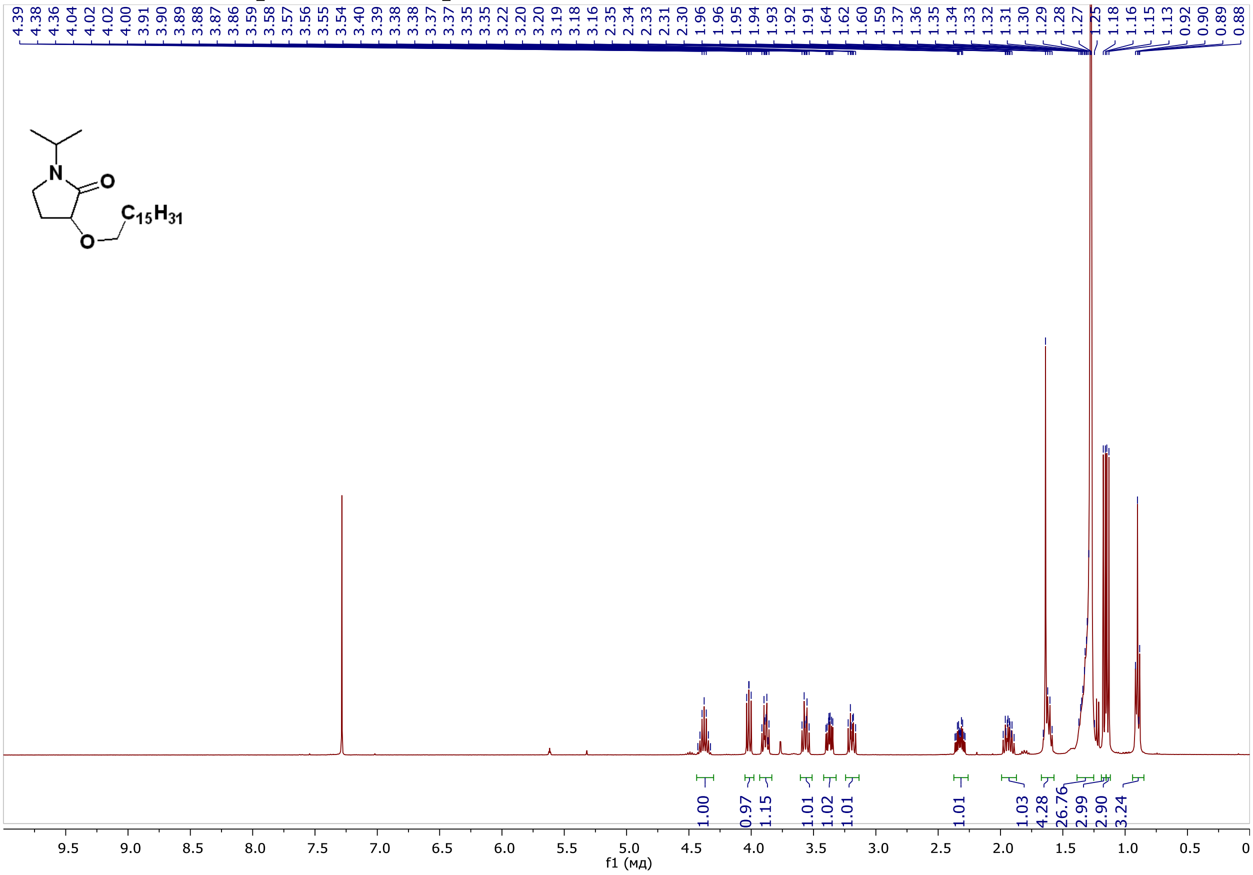

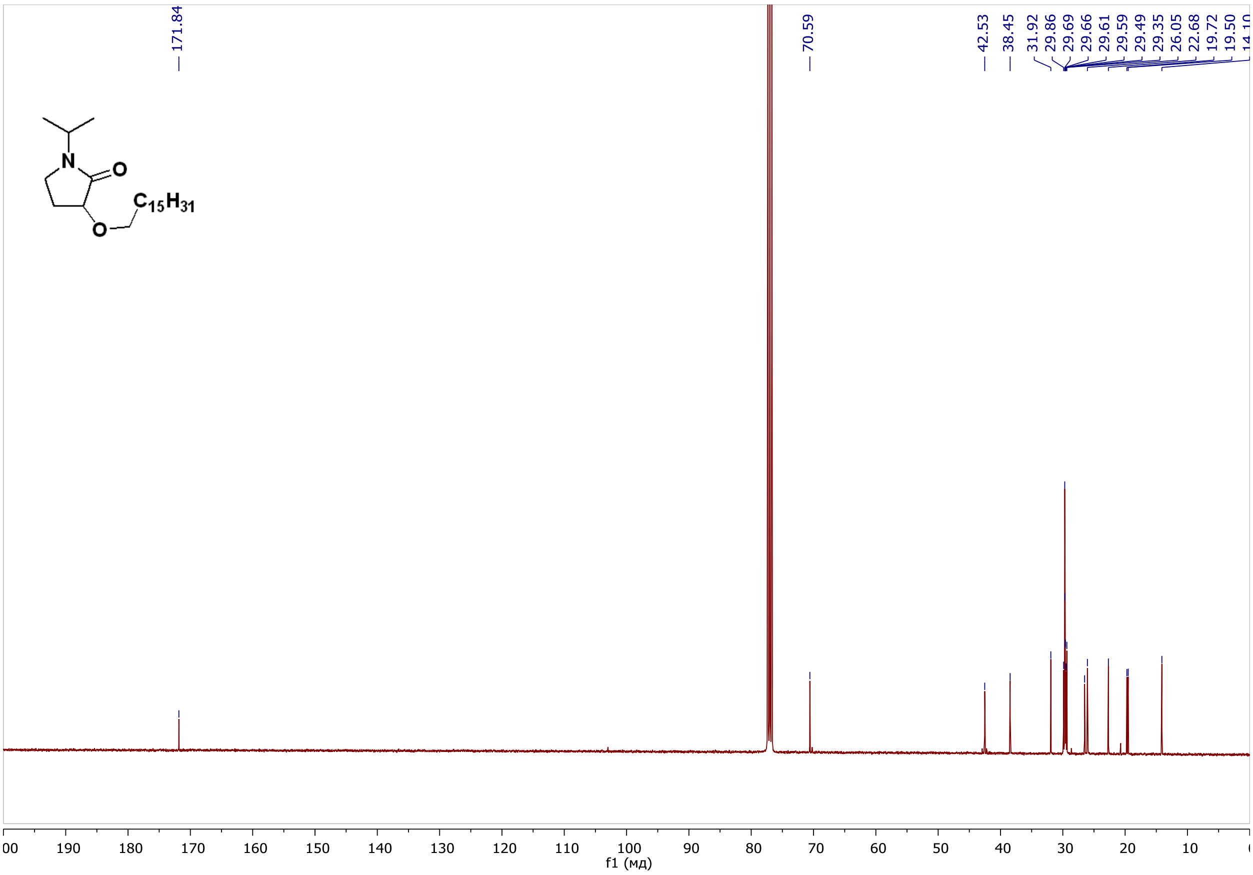

^{1}H and ^{13}C NMR spectra of compound 7j

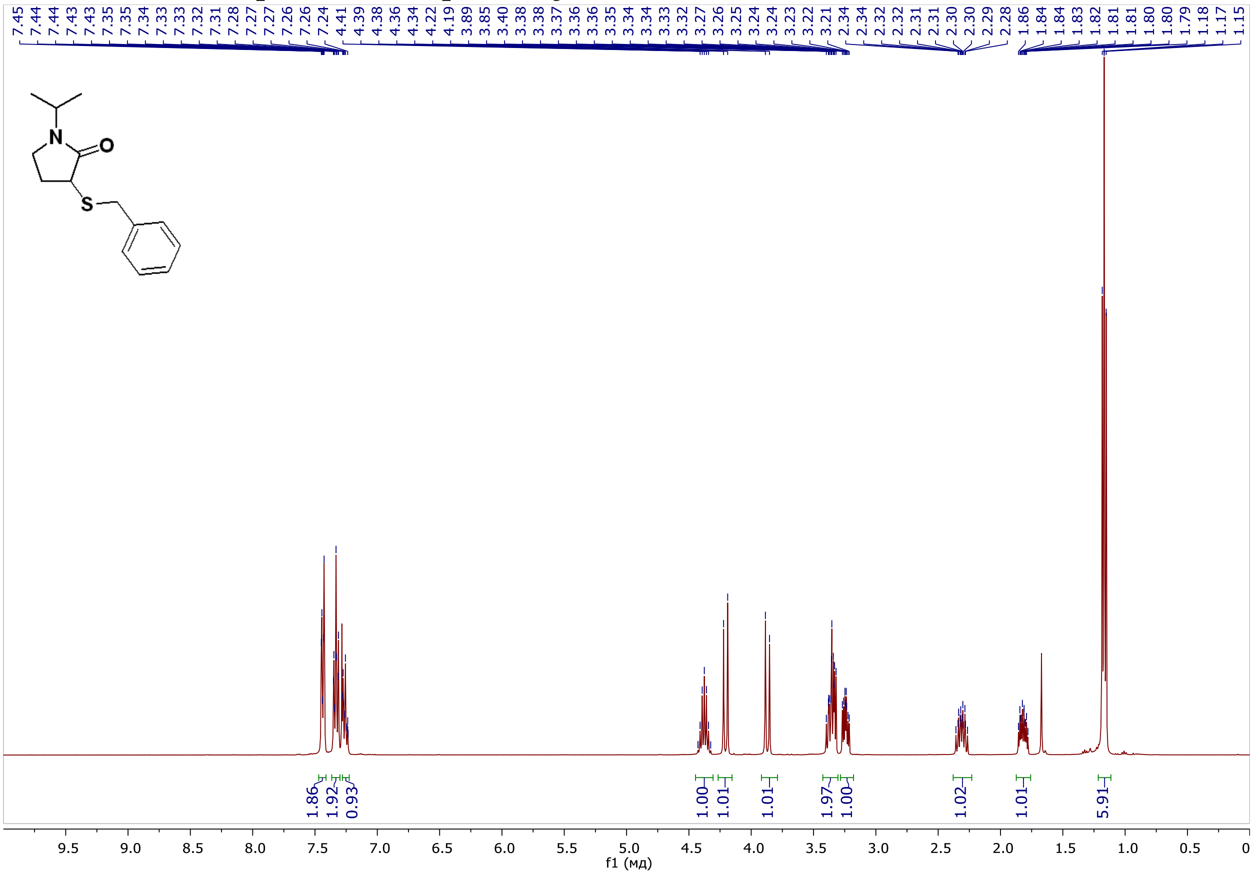

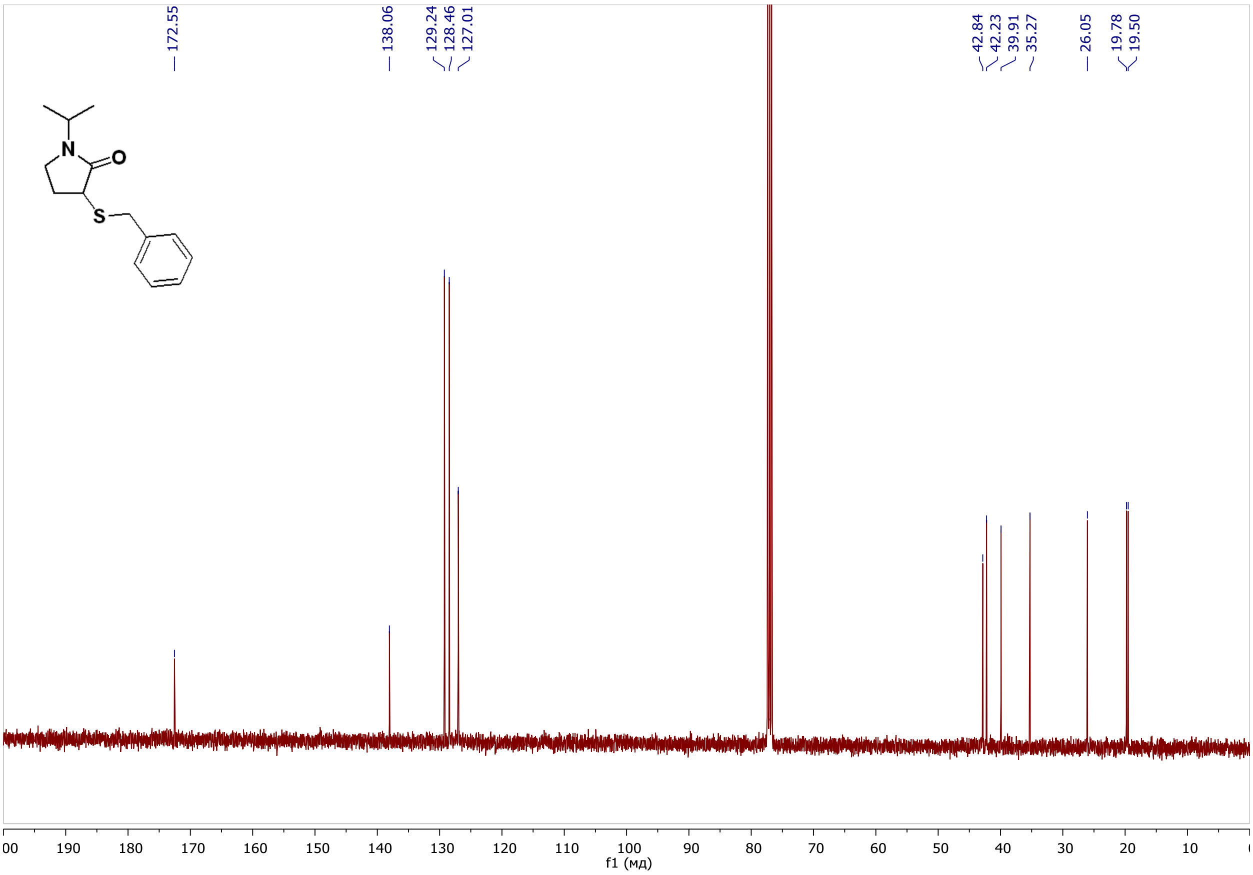

^{1}H and ^{13}C NMR spectra of compound 7k

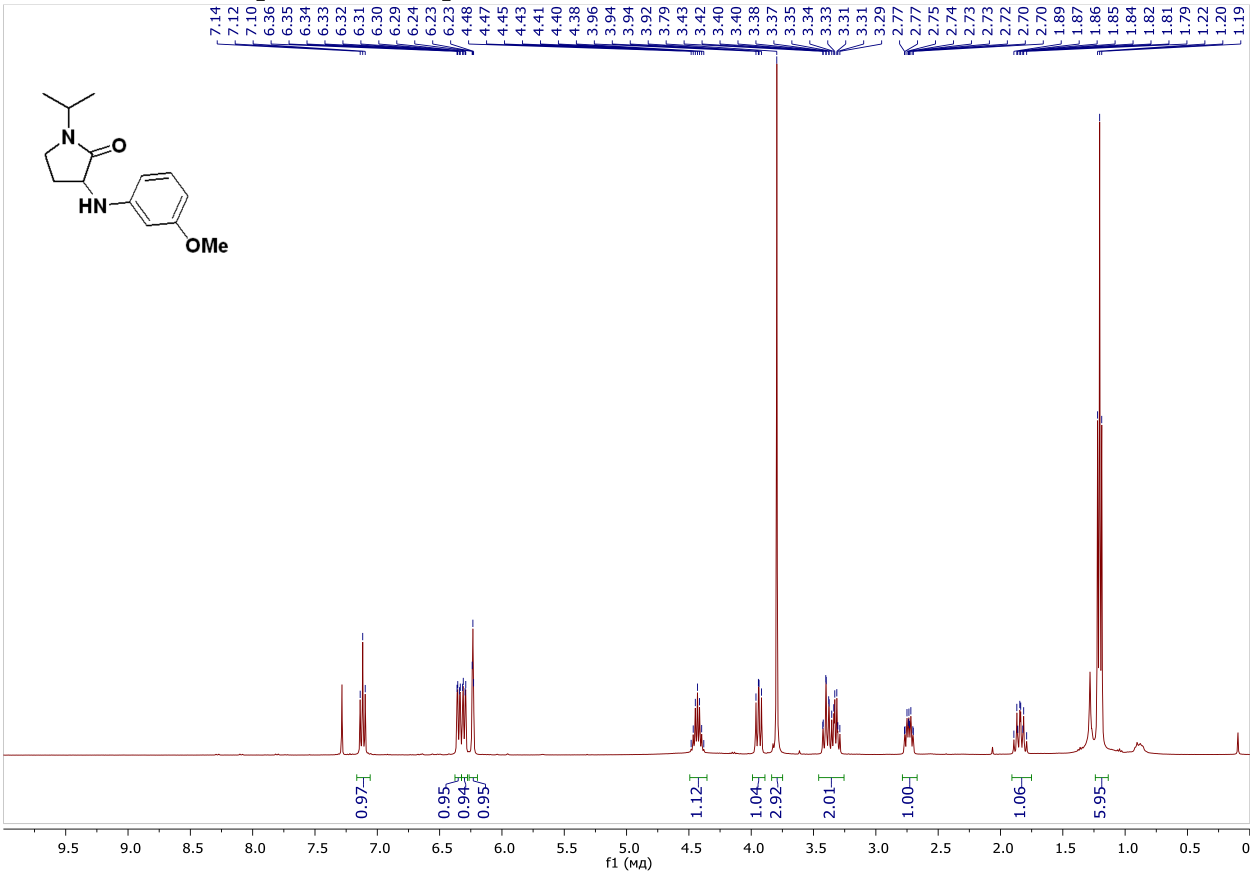

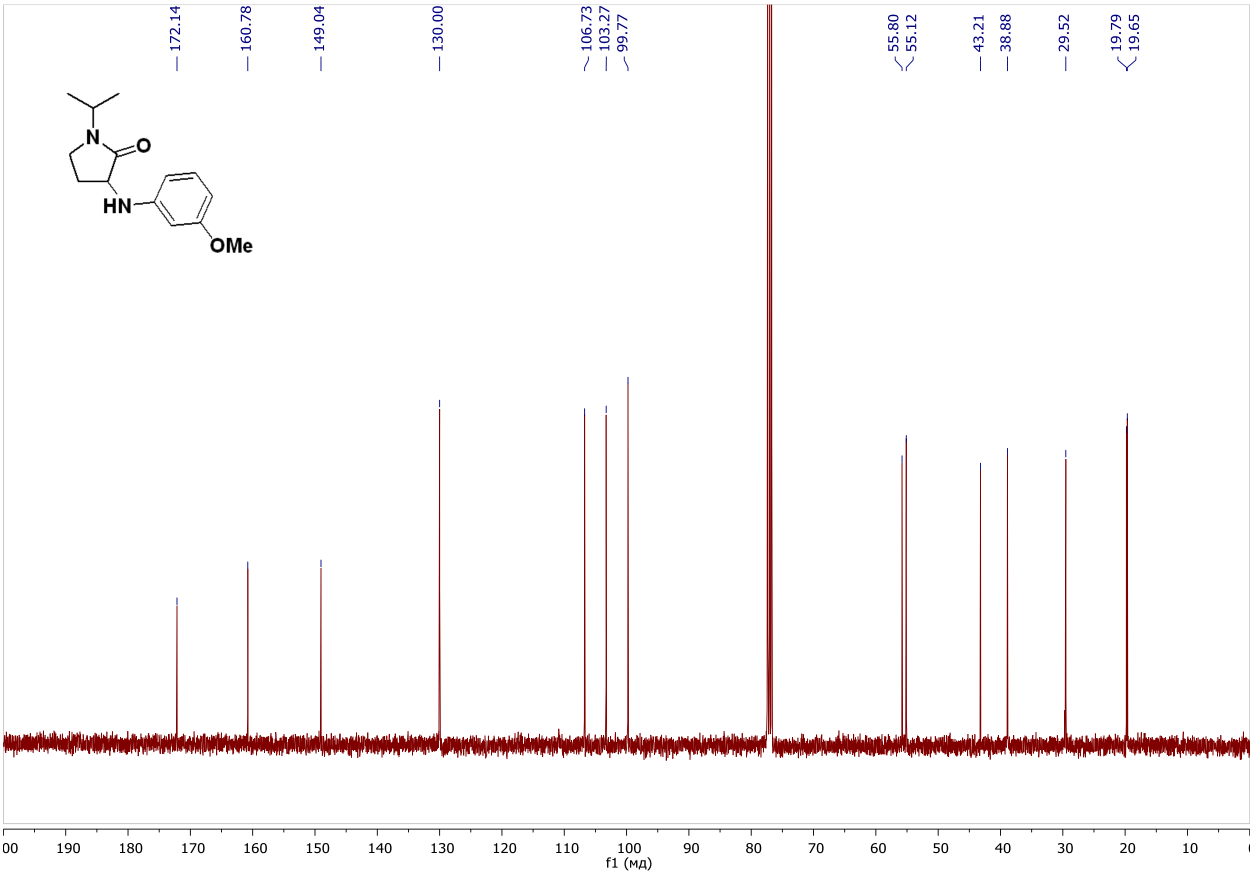

^{1}H and ^{13}C NMR spectra of compound 7l

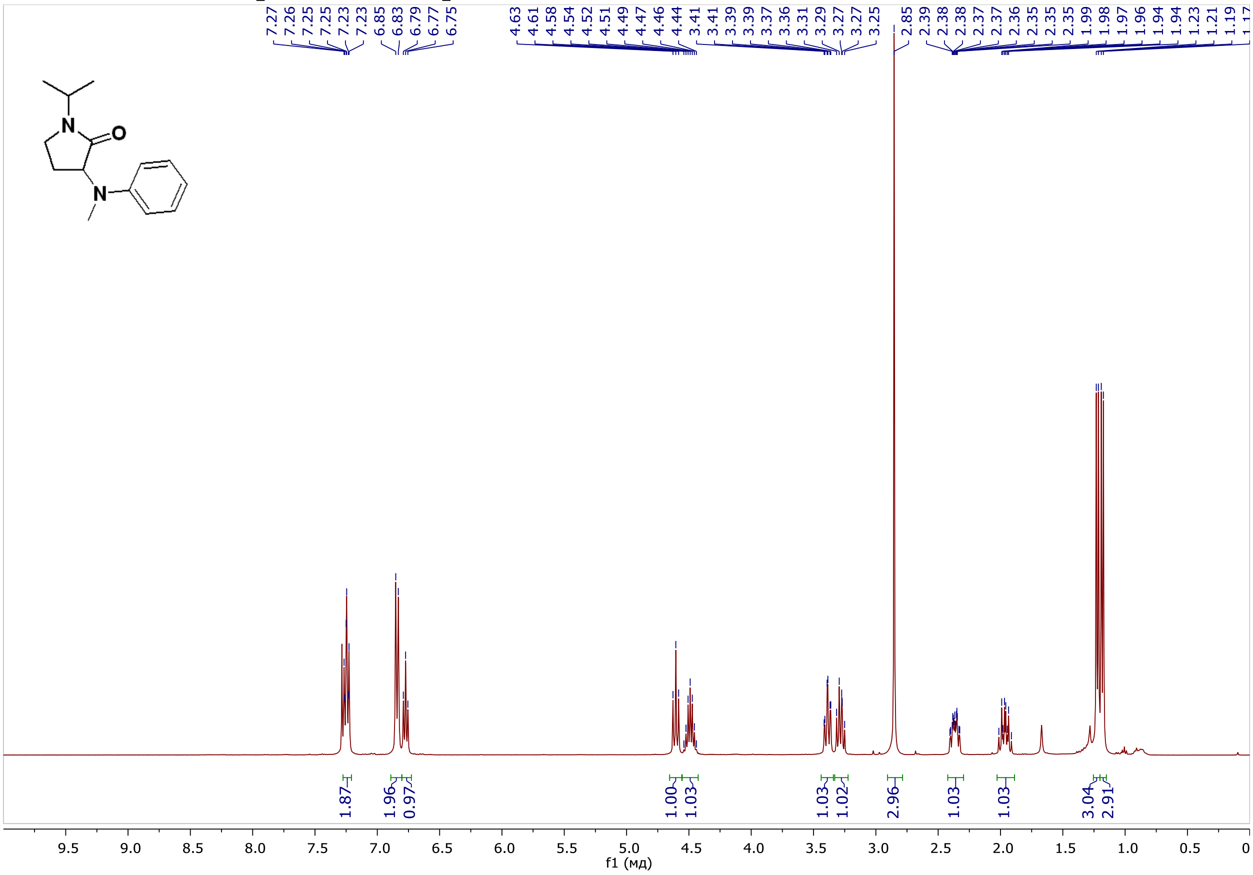

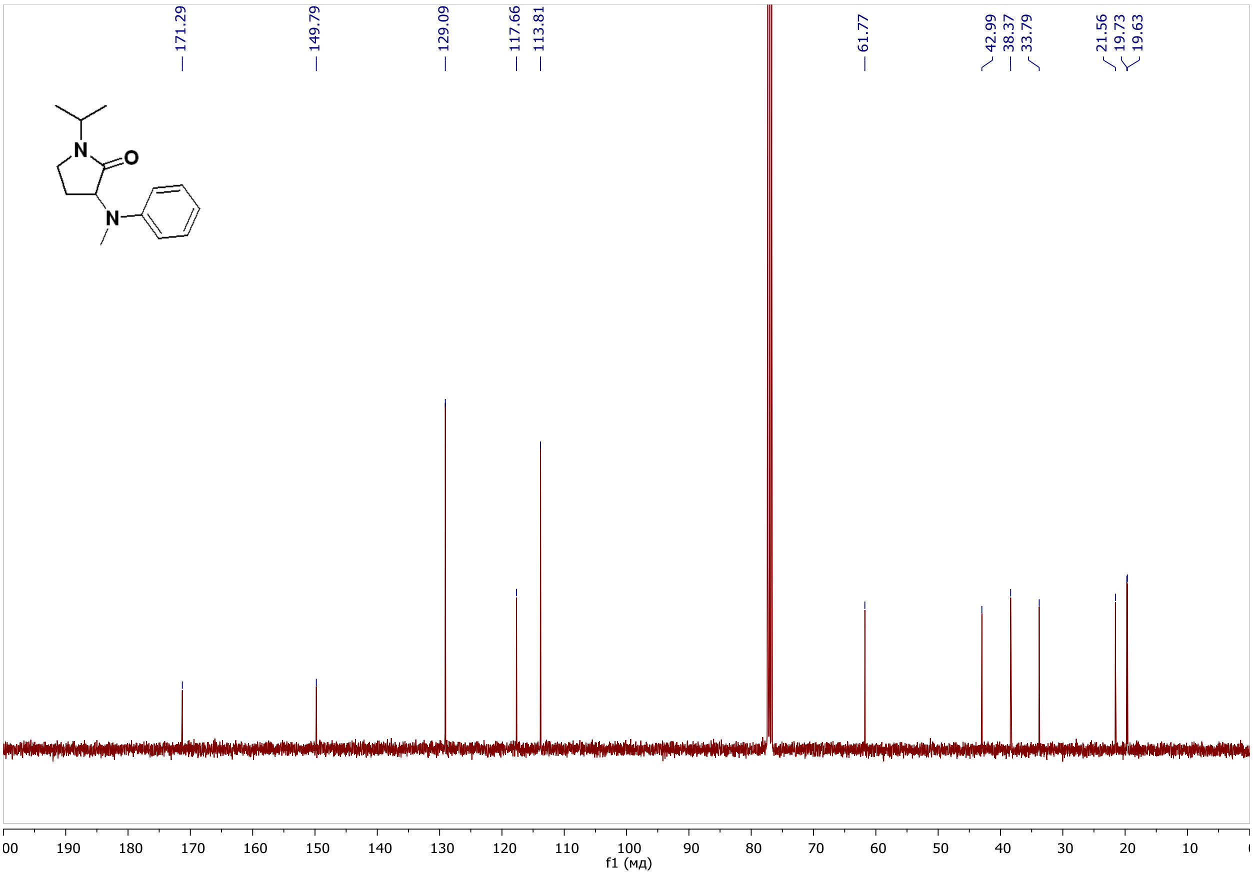

^{1}H and ^{13}C NMR spectra of compound 7m

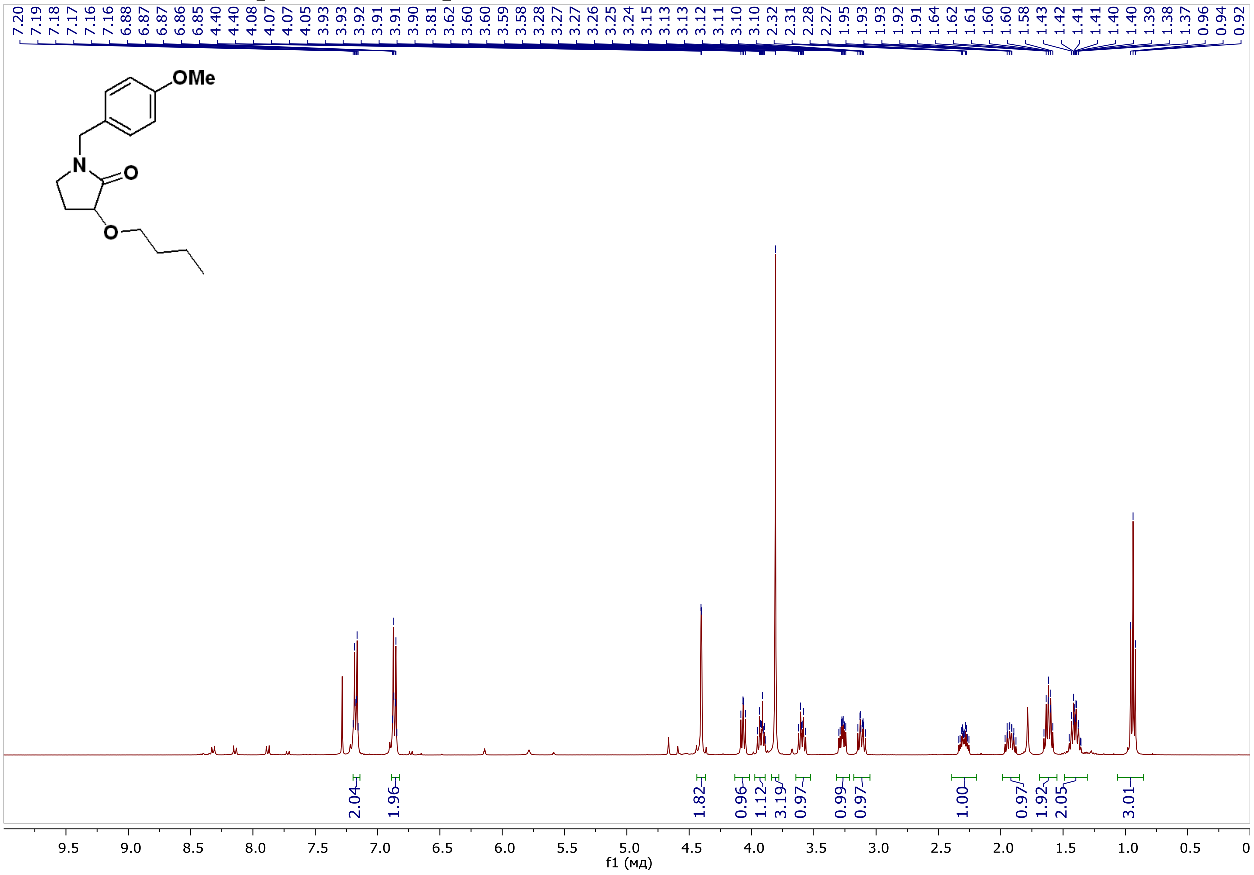

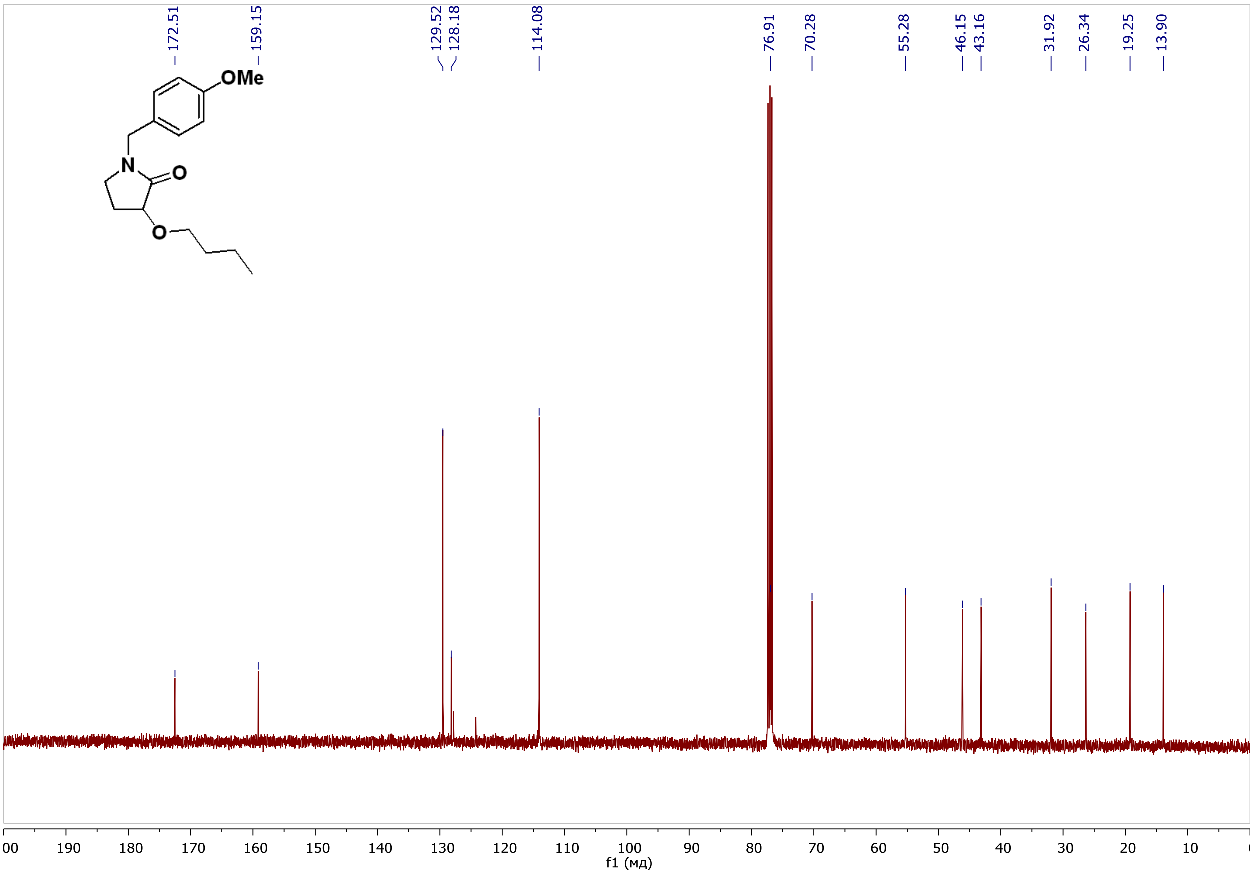

^{1}H and ^{13}C NMR spectra of compound 7n

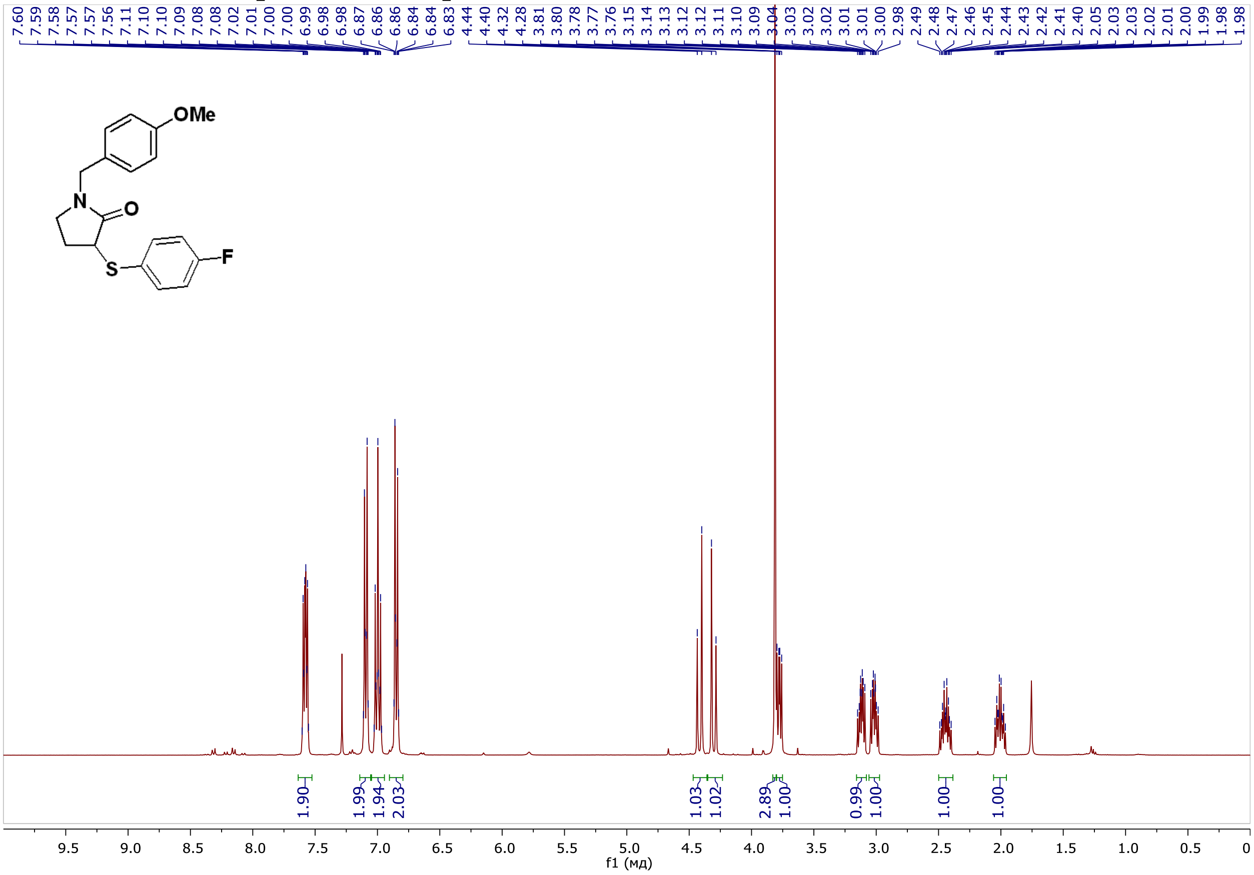

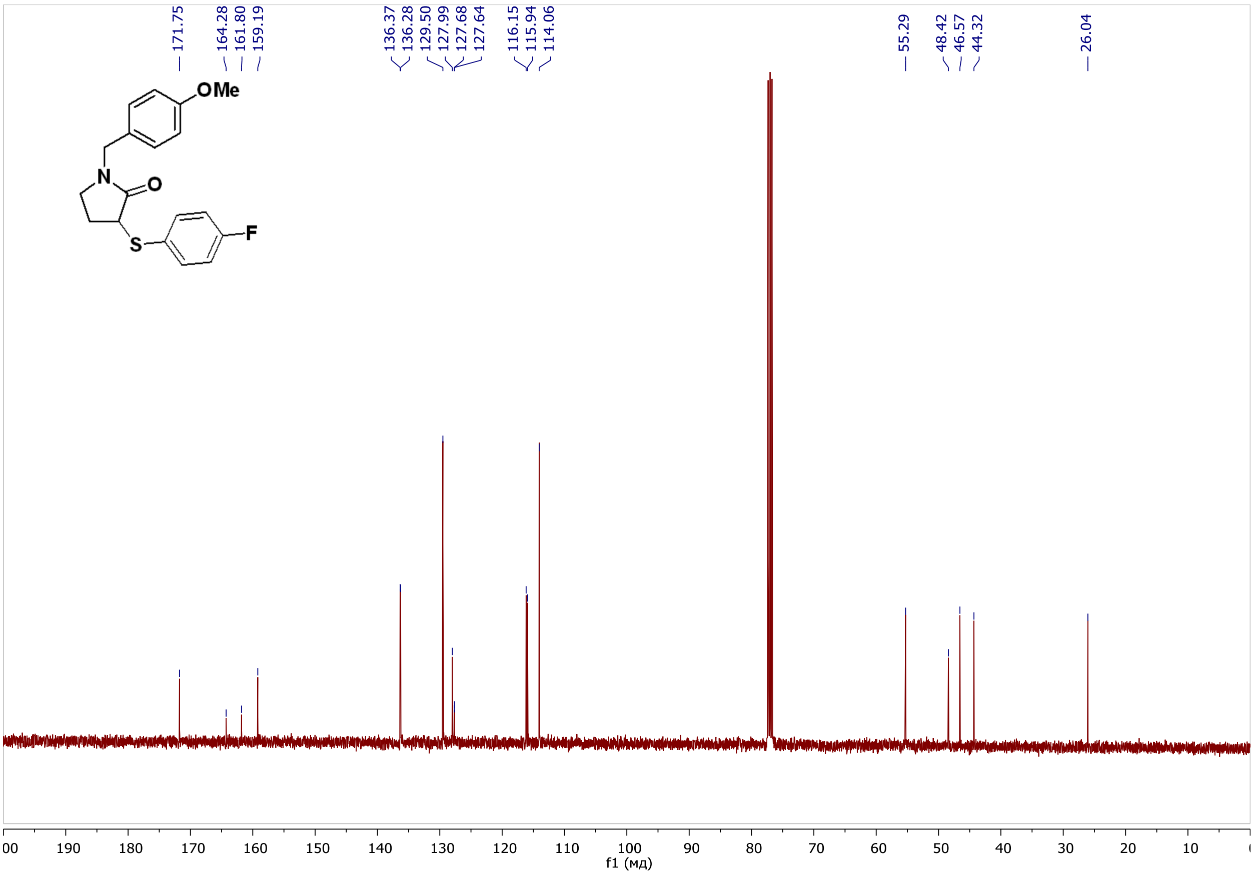

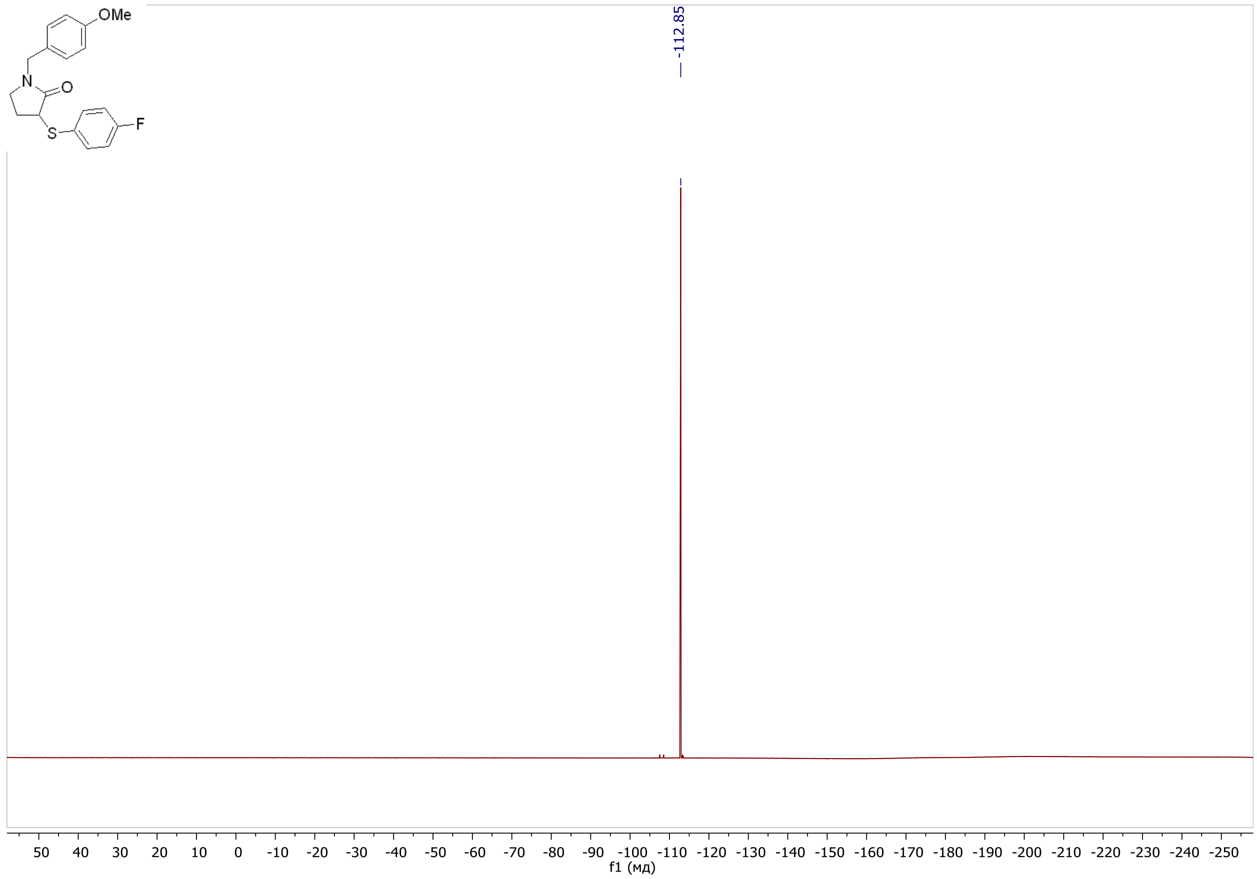
OMe
N
O
S
F
-112.85
50 40 30 20 10 0 -10 -20 -30 -40 -50 -60 -70 -80 -90 -100 -110 -120 -130 -140 -150 -160 -170 -180 -190 -200 -210 -220 -230 -240 -250
f1 (мд)

^{1}H and ^{13}C NMR spectra of compound 7o

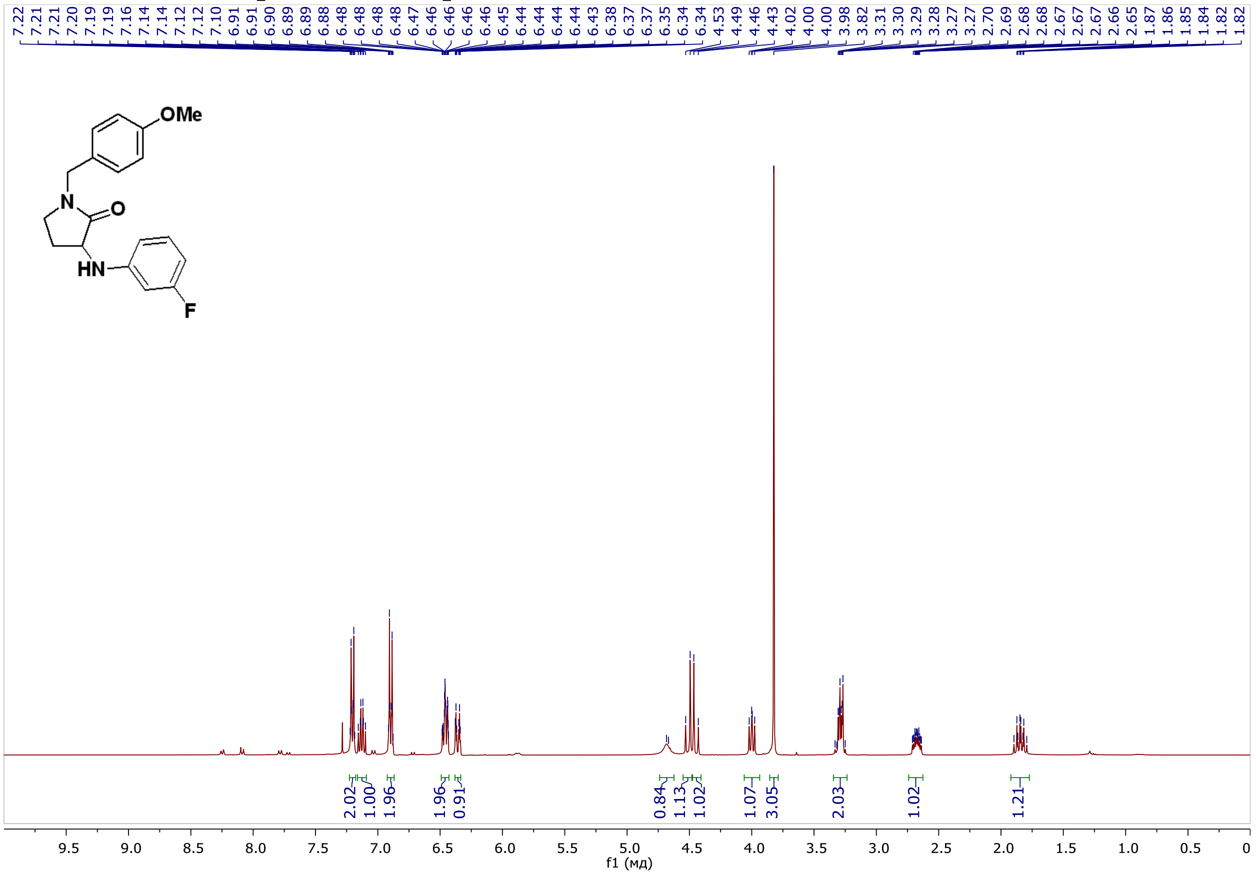

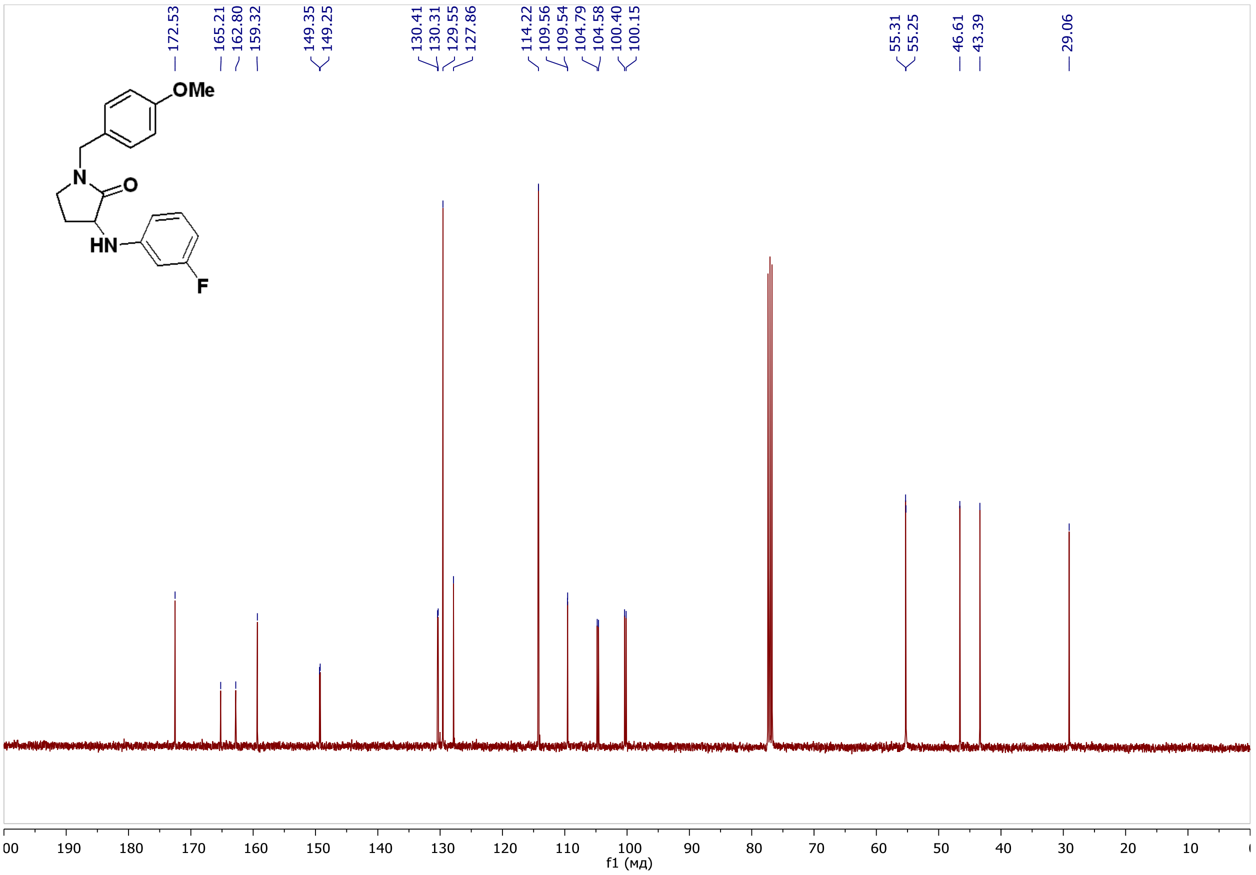

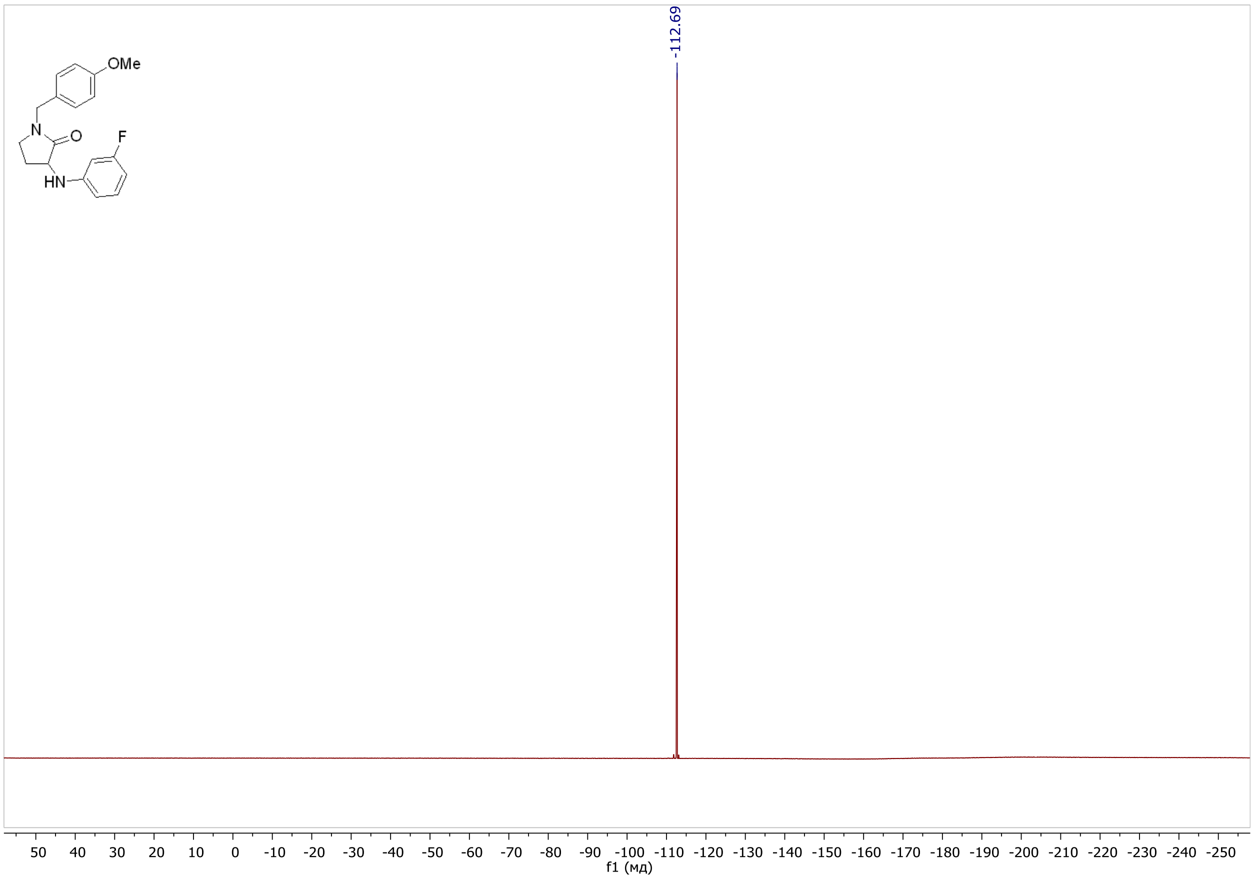

-112.69
OMe
N
O
F
HN
50 40 30 20 10 0 -10 -20 -30 -40 -50 -60 -70 -80 -90 -100 -110 -120 -130 -140 -150 -160 -170 -180 -190 -200 -210 -220 -230 -240 -250
f1 (мд)

^{1}H and ^{13}C NMR spectra of compound 8a

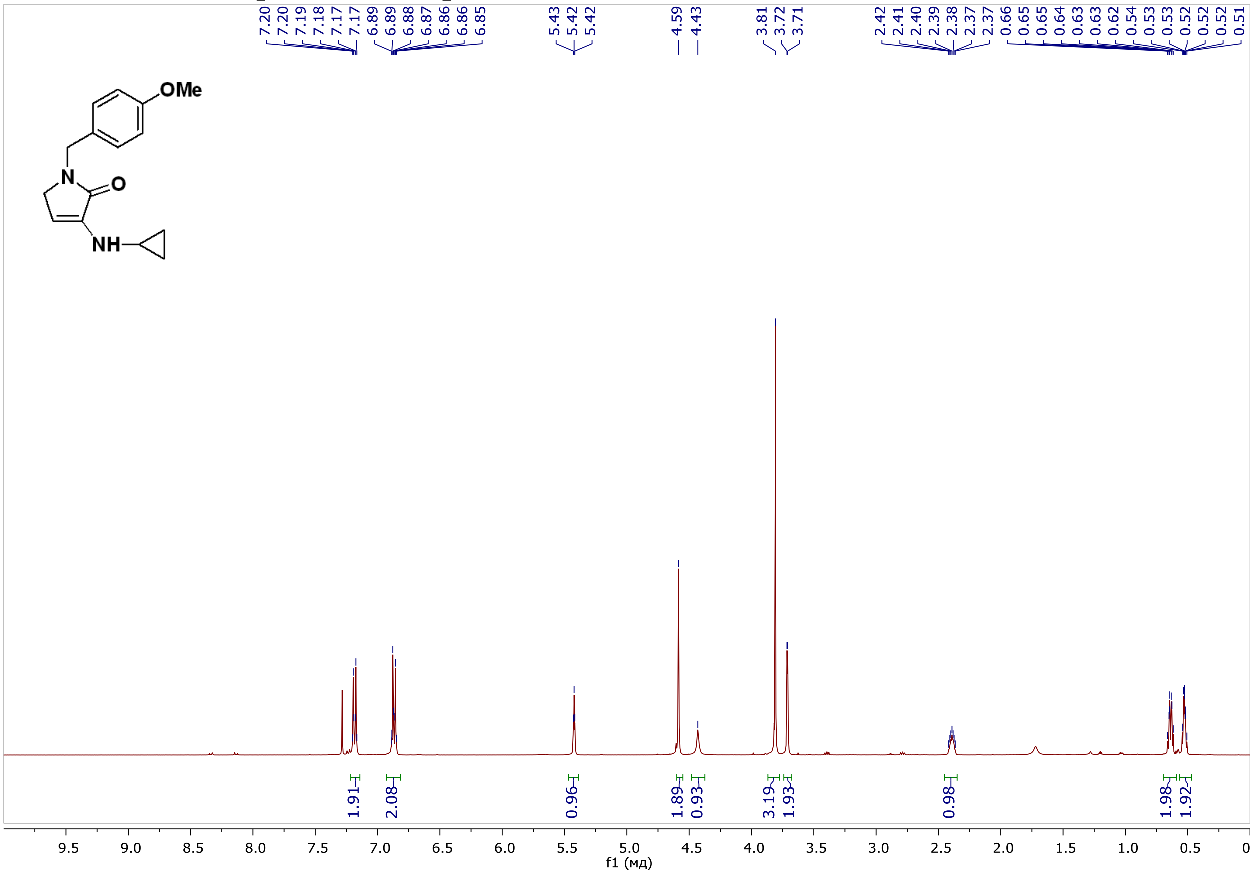

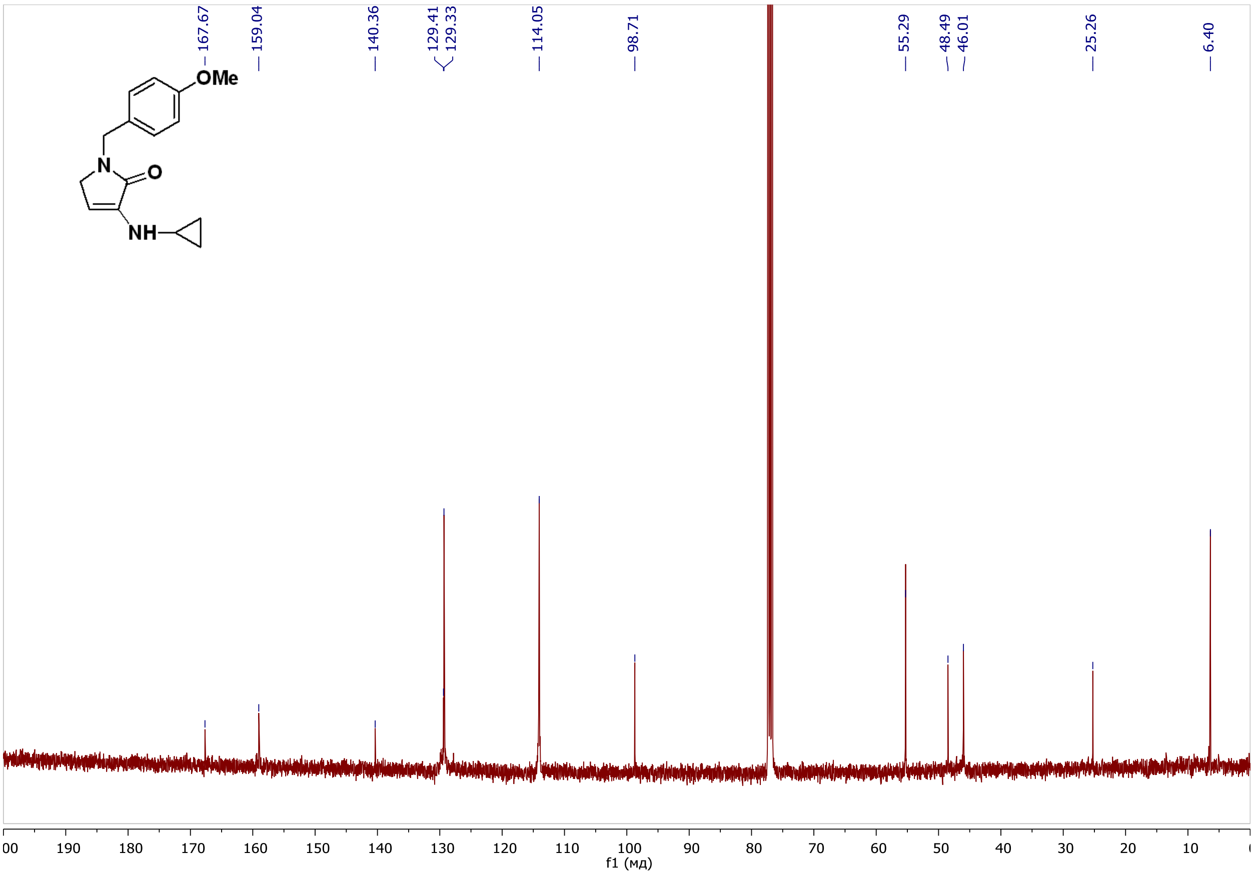

^{1}H and ^{13}C NMR spectra of compound 8b

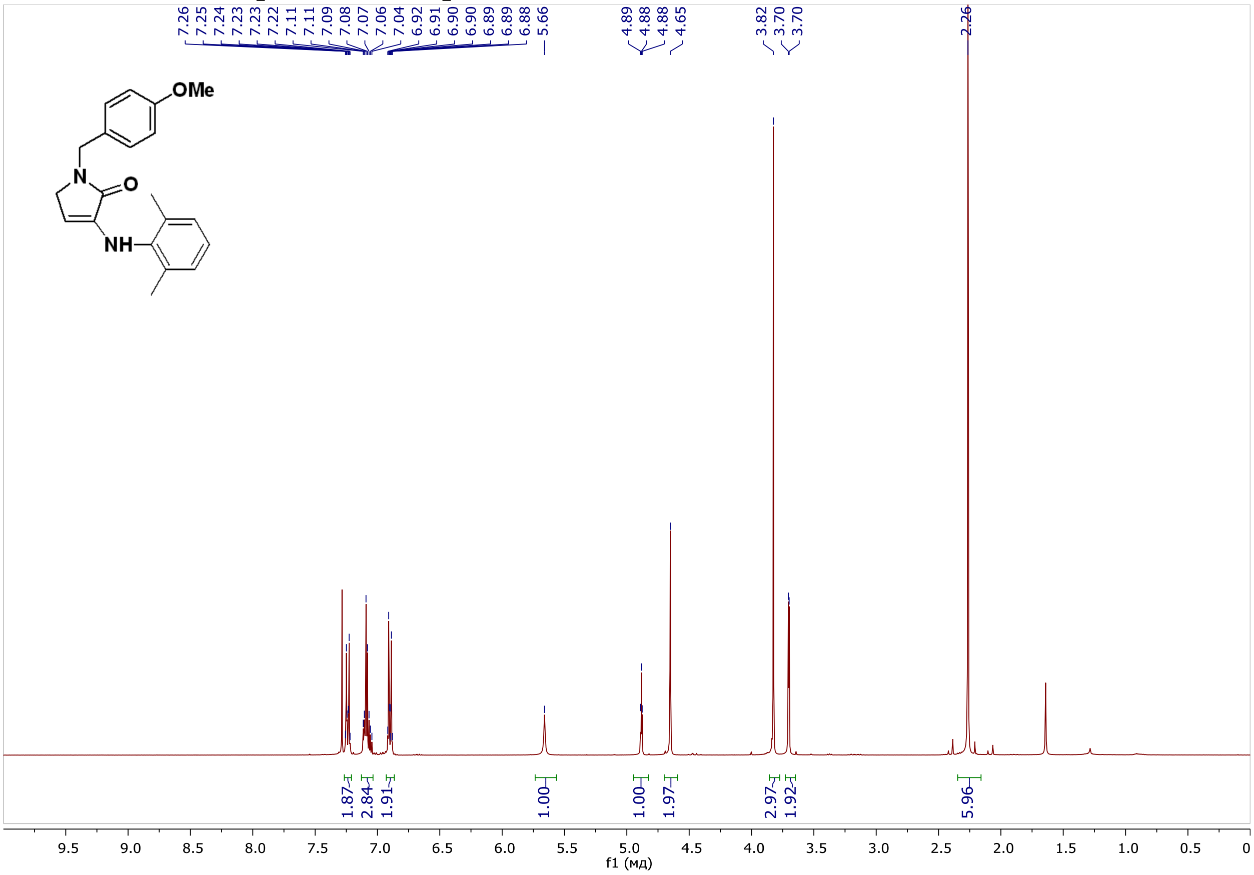

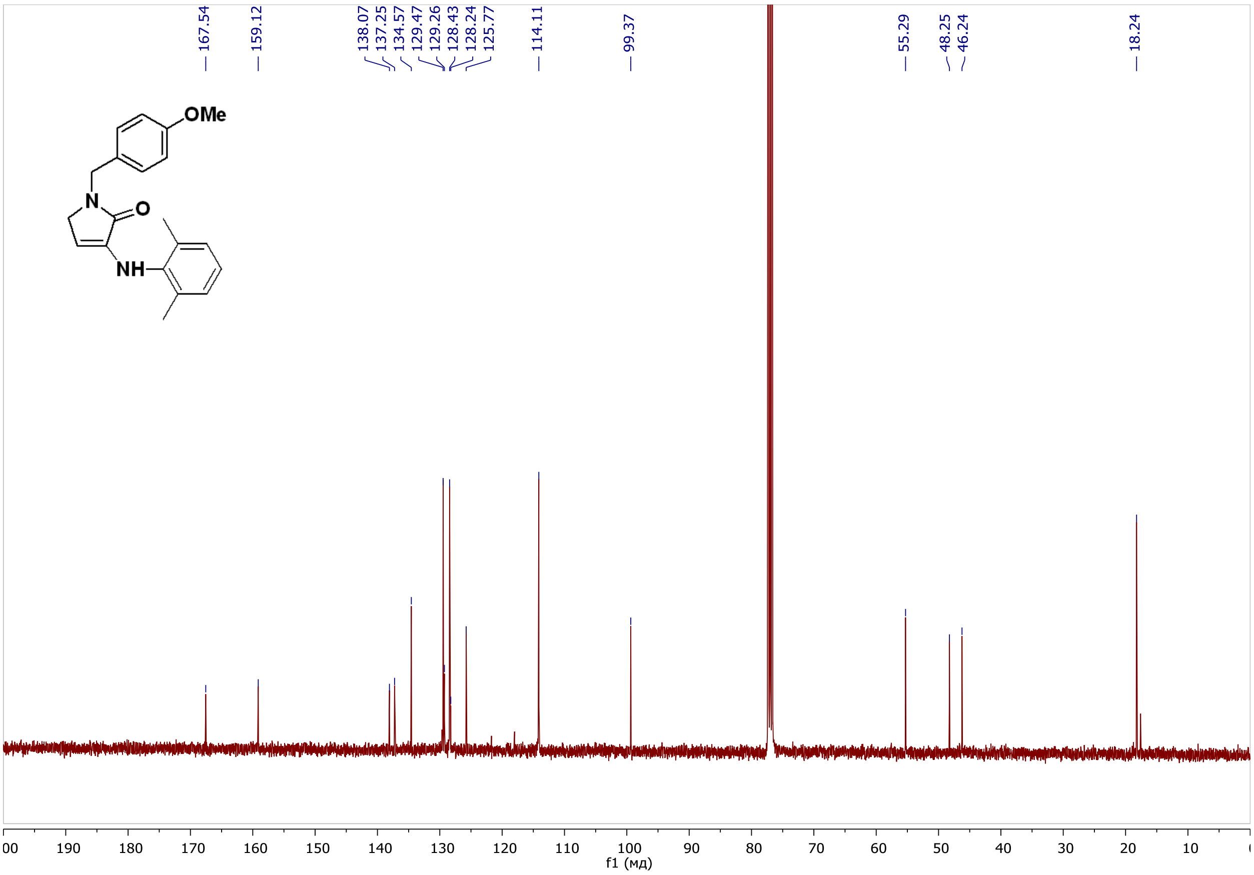

Supplement: File 1 — General experimental information, synthetic procedures, analytical data and NMR spectra for the reported compounds. [file Beilstein_J_Org_Chem-16-607-s001.pdf]
